# Propidium iodide staining underestimates viability of adherent bacterial cells

**DOI:** 10.1038/s41598-019-42906-3

**Published:** 2019-04-24

**Authors:** Merilin Rosenberg, Nuno F. Azevedo, Angela Ivask

**Affiliations:** 1 Laboratory of Environmental Toxicology, National Institute of Chemical Physics and Biophysics, Akadeemia tee 23, 12618 Tallinn, Estonia; 2 Department of Chemistry and Biotechnology, Tallinn University of Technology, Akadeemia tee 15, 12618 Tallinn, Estonia; 3 LEPABE - Laboratory for Process Engineering, Environment, Biotechnology and Energy; Department of Chemical Engineering; Faculty of Engineering, University of Porto, Rua Dr. Roberto Frias, 4200-465 Porto, Portugal

**Keywords:** Fluorescence imaging, Confocal microscopy, Microbiology techniques, Fluorescent dyes, Biofilms

## Abstract

Combining membrane impermeable DNA-binding stain propidium iodide (PI) with membrane-permeable DNA-binding counterstains is a widely used approach for bacterial viability staining. In this paper we show that PI staining of adherent cells in biofilms may significantly underestimate bacterial viability due to the presence of extracellular nucleic acids (eNA). We demonstrate that gram-positive *Staphylococcus epidermidis* and gram-negative *Escherichia coli* 24-hour initial biofilms on glass consist of 76 and 96% PI-positive red cells *in situ*, respectively, even though 68% the cells of either species in these aggregates are metabolically active. Furthermore, 82% of *E. coli* and 89% *S. epidermidis* are cultivable after harvesting. Confocal laser scanning microscopy (CLSM) revealed that this false dead layer of red cells is due to a subpopulation of double-stained cells that have green interiors under red coating layer which hints at eNA being stained outside intact membranes. Therefore, viability staining results of adherent cells should always be validated by an alternative method for estimating viability, preferably by cultivation.

## Introduction

Propidium iodide (PI) is widely used for bacterial viability staining, especially since Boulos *et al*. (1999) published the method^1^. PI can only cross compromised bacterial membranes and is therefore considered to be an indicator of membrane integrity. It stains DNA and RNA inside of dead cells or the ones with reversibly damaged membranes. For viability staining PI is usually coupled with a universal stain that crosses intact membranes and stains nucleic acids (NA) of all cells, thereby enabling to obtain total cell counts. One of the most common examples of such co-stain is SYTO 9. During co-staining with PI and SYTO 9, SYTO 9 can enter all cells regardless of their membrane integrity, bind to DNA and RNA and emit green fluorescence while PI can only enter cells with compromised membranes, bind to DNA and RNA and emit a red fluorescent signal. With higher affinity to bind DNA and in sufficient excess to SYTO 9, PI replaces SYTO 9, when both stains are exposed to the same DNA resulting in red fluorescent signal. As a result of coupling of those two DNA-binding and membrane permeability dependent stains red signals from cells are considered as “dead” and green signals as “alive”^1–3^. Although this principle is widely applied and proven to work well for an array of planktonic cultures, it has its limitations i.e. unequal SYTO 9 staining of viable and dead cells, incomplete replacement of SYTO 9 by PI or energy transfer during co-staining^2,4^. It has also been demonstrated that PI might in some cases provide false dead signals that could be associated with high membrane potential^5^, and that the staining result might be dependent on physiological processes other than membrane damage^6^. PI-based viability staining results do not always correlate with cultivability also due to the viable but not cultivable (VBNC) state of bacterial cells^7^ or cell clumping^8^. Despite its above-mentioned draw-backs, PI and SYTO 9 co-staining is also a widely used and suggested method in biofilm research^8–17^.

Another factor to consider when staining cells with NA-binding fluorophores is that NAs are not always only localized inside bacterial cells and surrounded by a membrane. For example, extracellular DNA (eDNA) can be present in planktonic cultures in specific growth phases^18^. During biofilm formation, eDNA mediates bacterial attachment to surfaces^19^, and it also plays a major role in mature biofilms. The importance of eDNA in biofilm formation has been proven by the fact that DNase I inhibits biofilm formation or detaches existing biofilm of several gram-positive and gram-negative bacterial species^20^. For the same reason, DNase is also proposed to be used as an anti-biofilm agent^21,22^. The presence of DNA from non-viable sources (eDNA and DNA from dead cells) has also introduced the need to use ethidium monoazide (EMA), propidium monoazide (PMA) or endonuclease (DNase I) treatment prior to viability assessment by quantitative polymerase chain reaction (qPCR)^23–25^. All the above-mentioned treatment agents, EMA, PMA as well as DNase I are intact membrane impermeable DNA-targeting compounds spatially targeting the same DNA as PI, depending on membrane integrity.

To get an overview whether the presence of eDNA in biofilms has been considered as a factor that may interfere with PI-based fluorescent staining, we performed a search in Scopus database for “biofilm” and “propidium iodide” and received 683 results while adding “extracellular DNA” or “eDNA” to the search decreased the number of results to 43 indicating that while PI is used for staining biofilms, possible presence of eDNA is generally not taken into account in this context. In the literature we can find that PI is also used for staining of eDNA^26,27^, but no clear quantitative proof about PI not being suitable for biofilm viability staining because of the presence of NA in biofilm extracellular matrix (ECM). More surprisingly, viability staining based on intact membrane impermeable DNA-binding stains like PI are occasionally used even while specifically studying eDNA^28^. Nonetheless, from some of the articles, hints of such threat can be found. For example, Gião and Keevil observed that some of *Listeria monocytogenes* biofilms in tap water and most of the old biofilms grown in rich media stained red with PI and SYTO 9 co-staining, but were cultivable and suspected red staining not to be indicative of dead cells but to be caused by eDNA^29^. From these sources it could be suspected that PI-based viability staining of biofilms, although commonly used, could be critically affected by eDNA and cause underestimation of biofilm viability. To address this possibility, we performed quantitative viability assessment of adherent cells using various staining and culture-based methods.

## Results

A combination of epifluorescence microscopy (EM), flow cytometry (FCM) and confocal laser scanning microscopy (CLSM) performed on propidium iodide (PI) and SYTO 9 stained adherent and harvested bacterial cells in parallel with culture-based methods was used to reveal whether staining of adherent bacteria with PI may underestimate their viability. Initial (24 h) biofilms of gram-negative *E. coli* K-12 wild-type substrain MG1655 and a gram-positive *S. epidermidis* type strain DSM-20044 were used for the experiments. *E. coli* MG1655 is widely used in molecular biology and capable of forming biofilm under both aerobic and anaerobic conditions^30–34^. *S. epidermidis* strains have well established biofilm forming properties similarly to *Staphylococcus aureus* and have been shown to produce eDNA^13,35^. The biofilms of these two bacterial strains on glass surfaces were formed in phosphate buffered saline (PBS) to rule out potential effect of osmotic stress on bacterial membranes and possibly consequently on viability staining outcome.

### Viability staining *in situ*

**Figure 1:**
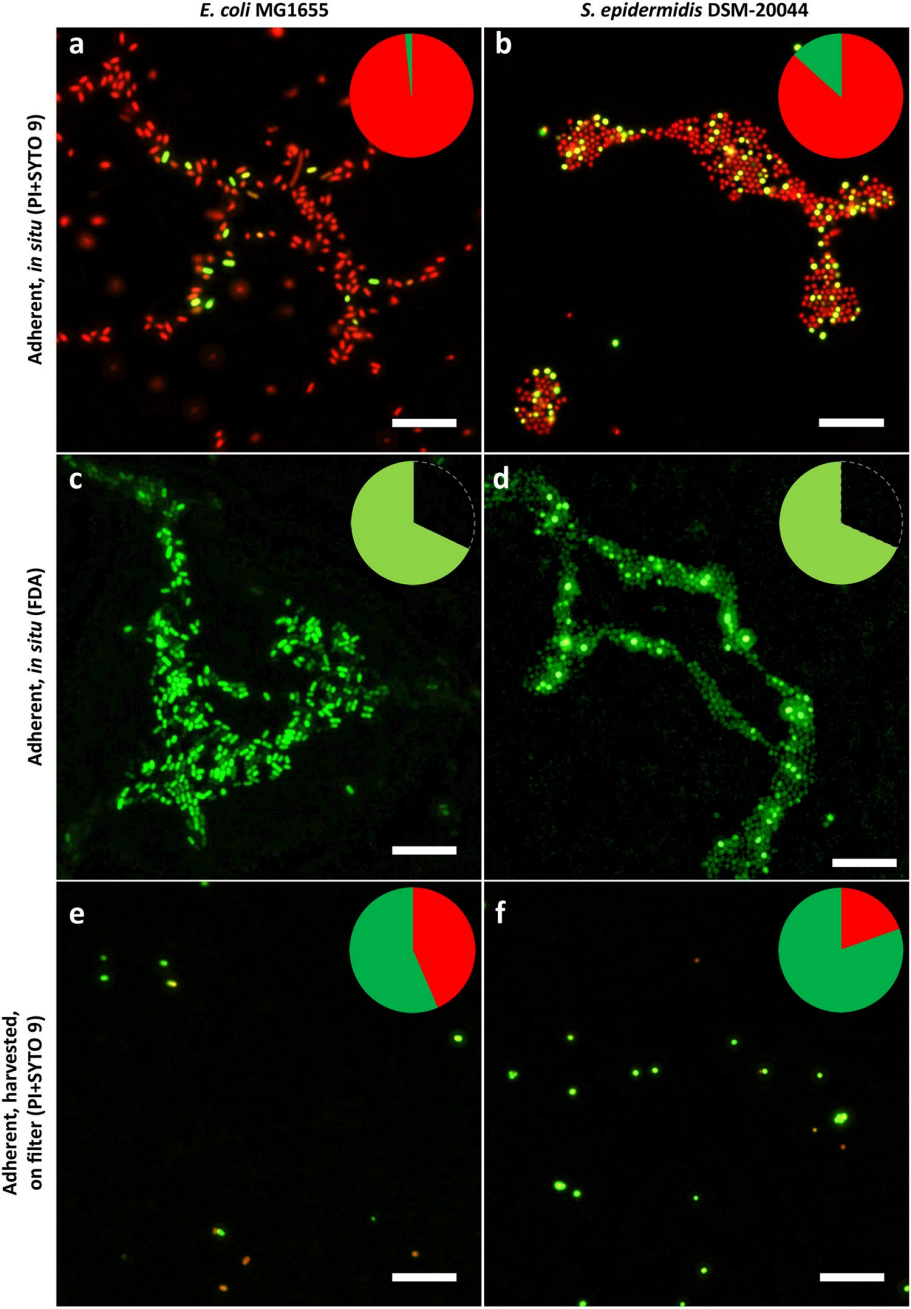
Epifluorescence microscopy images of adherent *E. coli* (**a,c,e**)  and S*. epidermidis* (**b,d,f**) viability staining. 24 h initial monolayer biofilm formed on glass in PBS stained *in situ* with propidium iodide (PI) and SYTO 9 (**a,b**), with fluorescein diacetate (FDA) (**c,d**) or harvested via sonication, stained with PI and SYTO 9 and collected on filter (**e,f**). Pie diagrams represent total cell count on surfaces with PI, SYTO 9 and FDA stained signal proportions marked in red, dark green and light green respectively. Scale bars correspond to 10 µm.

**Figure 2:**
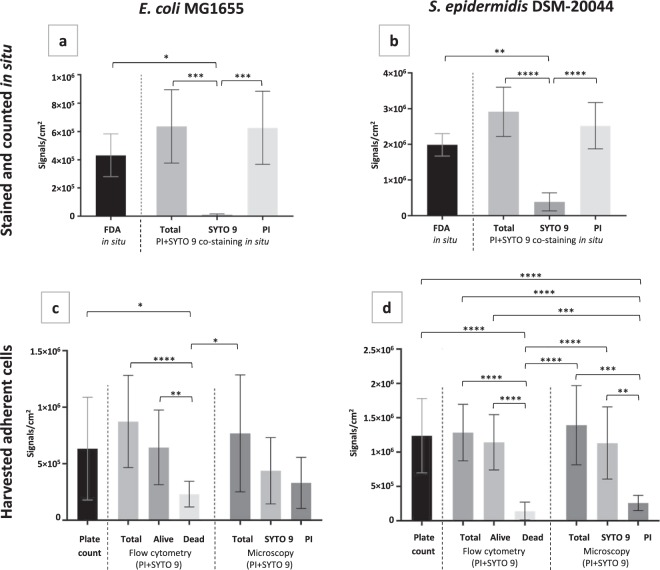
Comparison of multiple approaches to evaluate adherent cell viability in *E. coli* (**a,c**) and *S. epidermidis* (**b,d**) biofilms on surface *in situ* (**a,b**) or after harvesting via ultrasonication (**c,d**). 24 h initial monolayer biofilm formed on glass in PBS stained *in situ* (**a,b**) with propidium iodide (PI) and SYTO 9 or FDA followed by epifluorescence microscopy (EM) and signal counting or harvested (**c,d**) and cultivated for plate counts, co-stained with PI and SYTO 9 and analyzed by flow cytometry (FCM) or collected on filter followed by EM and signal counting. Cell counting results are presented as signals/cm^2^ where one signal counted corresponds to a single fluorescent cell or compact diplococcus (microscopy), a CFU (cultured plate count) or an FCM event. Live/dead gating of FCM signal populations was based on known proportions of viable and ethanol-killed planktonic bacteria. Mean and standard deviation of 4–6 independent values for *in situ* staining and filtering and 10–16 independent values for plate counts and FCM are shown and only statistically significant differences (p < 0.05) marked on graphs (“ “ > 0.05; * < 0.05; ** < 0.01; *** < 0.001; **** < 0.0001).

As can be seen on representative images (Fig. 1) and from quantitative data (Fig. 2), after PI + SYTO 9 co-staining, most adherent cells (96.35 ± 5.30% of *E. coli* and 75.69 ± 18.44% of *S. epidermidis* cells) in 24 h biofilm in PBS stained red with PI *in situ* (Figs 1a,b and 2a,b) while most (about 99%) planktonic cells from suspension above the respective biofilms stained green with SYTO 9 on a filter (Supplementary Fig. 1). This could normally be interpreted as simply showing the differences in the physiology of adherent and planktonic cells and different proportion of dead and alive cells indicating better viability of planktonic cells. However, decreased viability of adherent cells was not an expected result. Adherent cells presented biofilm-specific aggregation into microcolonies which is characteristic of viable initial biofilms. No toxic agent was used, and samples were rinsed before staining to ensure removal of loose dead planktonic cells. Also, the proportion of red-stained cells in the initial biofilms was surprisingly high. For example, using the same staining method, Wang *et al*. noted only a few dead cells among viable cells on a 24 h *E. coli* biofilm on silicone in PBS^36^. Starved biofilms incubated in PBS are more commonly used in oral health studies where most of the cells in biofilm tend to stain green similar to Zhu *et al*. reporting 76.7% viability of 24 h *Streptococcus mutans* biofilm on glass in phosphate buffer^9^. To exclude single stain effects, viable and ethanol-fixed biofilms were stained with PI, SYTO 9 and PI + SYTO 9 (Supplementary Figs 2 and 3). Single staining resulted in only red signals for PI and green signals for SYTO 9. Fixed samples stained with PI or PI + SYTO 9 showed only red cells. However, it could be observed that while single-stained fixed samples comprised of cells with similar PI or SYTO 9 intensity, variable signal intensities were observed for viable biofilms. Different binding affinity of SYTO 9 to viable and dead gram-negative bacteria is a known limitation of the method^4^. With adherent cells, we observed the same phenomenon also for gram-positive *S. epidermidis*.

As PI uptake by viable planktonic bacteria with increased membrane potential has been suggested by Kirchhoff and Cypionka^5^ and biofilms have been shown to demonstrate membrane potential fluctuations^37^ a control experiment deploying 3,3′-diethyloxacarbocyanine iodide (DiOC_2_(3)) and membrane potential eliminating carbonyl cyanide m-chlorophenylhydrazone (CCCP) pre-treatment was executed. No increased membrane potential signals described by Kirchhoff and Cypionka were observed for *in situ* DiOC_2_(3) staining and CCCP pre-treatment did not affect overall PI + SYTO 9 double- staining pattern of the biofilms (Supplementary Fig. 4), excluding the possibility of PI signals being caused by increased membrane potential.

To reveal the metabolic activity of *E. coli* and *S. epidermidis* in biofilms, we also stained the adherent cells with fluorescein diacetate (FDA), not a DNA-binding, but enzymatic activity indicative stain that emits green fluorescence after intracellular enzymatic cleavage^38^. It was observed that 67.91% *E. coli* and 68.30% *S. epidermidis* cells were metabolically active compared to *in situ* PI + SYTO 9 total counts (Figs 1c,d, 2a,b). Comparison of the results from staining the cells with FDA and PI + SYTO 9 showed that for both species of bacteria, there is a statistically significant difference in FDA and SYTO 9 signal counts (Fig. 2a,b) but there is no significant difference between FDA and PI or total (PI + SYTO 9) signal counts. On the assumption that dead cells are not metabolically active, and starvation may even cause underestimation of viable cell count based on FDA staining, this result sharply contradicts PI + SYTO 9 viability staining results. From these results demonstrating that most of the cells on glass surfaces are metabolically active and stain with PI while a minority of presumably viable cells stain with SYTO 9 only it can be concluded that SYTO 9 signal count significantly underestimates viability and PI signal count significantly overestimates dead cell count as a result of PI + SYTO 9 co-staining *in situ*. Counting possible weak background FDA signals from dead cells was ruled out as FDA-staining of ethanol-fixed biofilms did not produce observable signals (Supplementary Fig. 5). However, neither membrane integrity nor enzymatic activity, especially when incubated in a nutrient-poor environment, can truly indicate the reproduction capability of the cells. This can only be measured by cultivation-based methods.

### Viability staining and cultivability of harvested cells

Adherent bacteria were harvested from the surfaces via ultrasonication optimized to acquire the maximum number of viable cells (Supplementary Fig. 6) and plated or stained with PI and SYTO 9 and analyzed by flow cytometry (FCM) or collected on filter followed by epifluorescence microscopy (Figs 1e,f, 2c,d). Of the 96.35 ± 5.30% *in situ* PI-positive *E. coli* cells, only 43.50 ± 5.30% were PI-positive after harvesting, subsequent staining and collection on filter, and only 27.76 ± 9.61% of those cells could be assigned to the “dead” gate in FCM, based on ethanol-killed planktonic cells. Similarly, of the 75.69 ± 18.44% *in situ* PI-positive *S. epidermidis* cells, only 19.56 ± 8.93% were PI-positive after harvesting, staining and collection on filter, and only 11.07 ± 10.70 those cells could be assigned to the “dead” gate in FCM. This result showing increased fraction of SYTO 9 stained cells after harvesting of adherent cells via ultrasonication compared with adherent cells *in situ* (Fig. 1a vs 1e; 1b vs 1f) was rather surprising. One would expect that ultrasonication does not increase but rather decreases cellular viability due to physical damage as longer ultrasonication durations resulted in decreased planktonic cell viability as well as decreased viable yield of adherent cells (Supplementary Fig. 6). However, due to the seemingly reversed red to green ratio after ultrasonication we hypothesized that this treatment affects the staining of viable cells with PI. One of the possible explanations for that was partial removal of eNA containing ECM from adherent cells. Indeed, ultrasonication is a technique that is commonly used for ECM extraction^39,40^. Removal of eNA and false dead signals along with ECM was further confirmed by cultivating the harvested bacteria. Following the PI + SYTO 9 staining principle, plate counts could be expected to be smaller than the number of SYTO 9 signals from *in situ* staining due to possible cell aggregates forming only one colony but yielding several signals counted. On the contrary, compared to total signal counts from harvested and PI + SYTO 9 stained samples at least 82.43% of *E. coli* and 89.02% of *S. epidermidis* cells were cultivable and formed colonies on nutrient agar.

There was no statistically significant difference in plate counts of biofilm harvested cells, FCM total event counts FCM “alive” event counts, and SYTO 9 counts of harvested, PI + SYTO 9 stained and filtered samples for neither species, indicating that the majority of the harvested cells are truly viable (Figs 2c,d) and the fact that they stained red with PI in *in situ* biofilms was indeed an artifact, most likely due to the presence of eNA in the biofilm matrix.

Of the approaches used for harvested cell viability assessment, FCM proved to be a quicker and less elaborate method than filtering stained samples and counting fluorescent signals from microscopy images but gating harvested sample signals in FCM proved to be problematic. FCM alive and dead gates were based on viable and ethanol-killed planktonic cultures but unlike planktonic samples from the same test system, ultrasonicated biofilm samples had much higher noise level and less defined and/or shifted “alive” signal populations (Supplementary Fig. 7) easily explained by partial ECM removal during harvesting and resulting double-staining of viable bacteria to various degrees in contrast to more strictly PI-defined “dead gate”.

The fact that viability estimate based on *in situ* PI-staining was significantly lower than the ones based on *in situ* FDA staining or harvested cell plate count (Table 1) suggested that eNAs could indeed play a major role in false “dead” PI-staining of biofilm bacteria *in situ*. To further confirm the hypothesis, confocal microscopy was used to better visualize the PI and SYTO 9 co-stained bacterial biofilms.

**Table 1:** Viability estimates (%)* of 24 h biofilms acquired with different methods.

Species	*In situ* PI + SYTO 9	*In situ* FDA vs PI + SYTO 9 total count	Harvested, PI + SYTO 9, on filter	Harvested, PI + SYTO 9, flow cytometry	Harvested, plate count vs PI + SYTO 9 total count on filter
*E. coli*	3.65 ± 5.30	67.91	56.50 ± 5.30	77.20 ± 9.60	82.43
*S. epidermidis*	24.31 ± 18.44	68.30	80.44 ± 8.93	88.90 ± 10.70	89.02

*Mean and standard deviation of 4–6 independent values for *in situ* staining and filtering and 10–16 independent values for plate counts and FCM are shown. Percentages calculated as ratios of mean values are presented without standard deviations.

### Confocal laser scanning microscopy (CLSM) of PI + SYTO 9 stained biofilms

**Figure 3:**
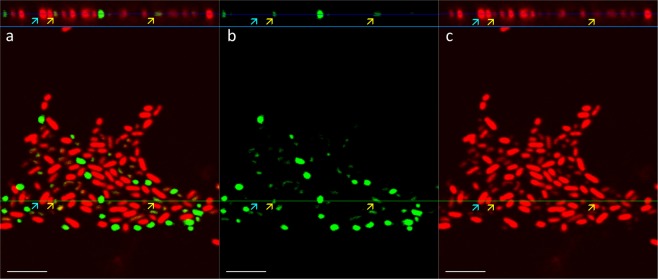
Confocal laser scanning microscopy (CSLM) images of 24 h *E. coli* biofilm co-stained with propidium iodide (PI) and SYTO 9: vertical and horizontal cross-sections in multichannel (**a**), green channel (**b**) and red channel (**c**) view. Dead cells stained with PI are indicated with cyan and viable cells double-stained with PI and SYTO 9 with yellow arrows. Scale bars correspond to 5 µm. Single images of the Z-stack are available in Supplementary Album 1.

**Figure 4:**
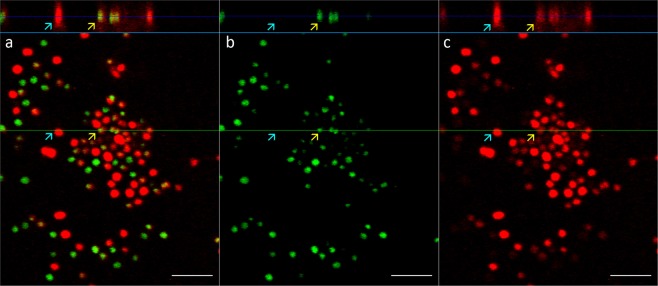
Confocal laser scanning microscopy (CSLM) images of 24 h *S. epidermidis* biofilm co-stained with propidium iodide (PI) and SYTO 9: vertical and horizontal cross-sections in multichannel (**a**), green channel (**b**) and red channel (**c**) view. Dead cells stained with PI are indicated with cyan and viable cells double-stained with PI and SYTO 9 with yellow arrows. Scale bars correspond to 5 µm. Single images of the Z-stack are available in Supplementary Album 1.

CLSM cross-sections of monolayer biofilms revealed overlapping PI and SYTO 9 signals of *E. coli* (Fig. 3) and *S. epidermidis* (Fig. 4) creating a wider diffuse red PI corona around SYTO 9 signals except for the most intensely red cells that lacked green signal and could presumably be true dead signals. It must be noted that the result was seriously affected by vertical resolution limit of CLSM due to bacterial cell size, especially for *E. coli*. However, it was still possible to bring light to the fact that a large proportion of the cells of both species demonstrated double-staining with green interiors under red PI-stained exteriors. This double-staining was only characteristic of viable biofilms and also not a single stain effect, as co-stained ethanol-fixed biofilms lacked green signals with the same CLSM setup and biofilms monostained with PI or SYTO 9 only produced signal in their respective emission channels (Supplementary Figs 8 and 9). Full width at half maximum (FWHM) measurements of CLSM cross-sections of non-saturated cellular signals confirm that red signals from double-stained cells of both species are significantly wider than green signals (Supplementary Fig. 10).

As suspected from the comparison of staining of cells with PI + SYTO 9 *in situ* and after ultrasonication, CLSM confirms that PI really does stain cells externally and is therefore not indicative of viability but produces false dead signals under these experimental conditions. Unfortunately, the CLSM resolution limit does not allow to confirm PI exclusion from cell interiors. To compare the staining pattern of PI + SYTO 9 to SYTO 9 co-staining with membrane or membrane-associated ECM components, we combined SYTO 9 with CellMask Orange (CMO), Nile red (NR) and Congo red (CR). NR + SYTO 9 staining resulted in the strongest signals of both NR and SYTO 9 (Supplementary Fig. 11) with a similar red corona around green cell interiors apparent in CLSM as was observed for PI + SYTO 9 staining (Fig. 4). CMO staining resulted in weaker signals for *S. epidermidis* (Supplementary Fig. 11) and was not usable for *E. coli* but demonstrated a similar red corona around green *S. epidermidis* cells as PI + SYTO 9 and NR + SYTO 9. Due to imaging at the CLSM resolution limit, membrane signals (NR, CMO) can also be seen inside the cells similarly to PI signals on Figs 3 and 4. Expected extracellular DNA signal (SYTO 9) outside the membranes similar to extracellular PI signal in case of PI + SYTO 9 co-staining is lost in these images (Supplementary Fig. 11) possibly due to limited dynamic range and loss of weaker signals in single channel SYTO 9 acquisition as opposed to individually adjustable separate channels for intracellular SYTO 9 and extracellular PI signal acquisition (Figs 3 and 4).

CR is an amyloid stain fluorescing red when bound to bacterial surface-associated amyloid fibrils (SAFs)^41^. CR signals appeared too weak and quickly bleaching to use in CLSM, but images could be attained in epifluorescence microscopy using long exposures. Interestingly CR + SYTO 9 staining pattern in epifluorescence microscopy (Supplementary Fig. 12) appears similar to that of PI + SYTO 9 (Fig. 1a,b) with some cells lacking red signals and staining green with SYTO 9 while most present red CR signals of variable intensities. It is also evident that SYTO 9 signal is completely masked by more intense CR signals, similarly to PI + SYTO 9 with the significant difference that CR + SYTO 9 eliminates competing for DNA binding.

To further prove the role of eDNA in PI-staining, we also attempted to treat biofilms and similar amount of planktonic cells with DNase I, remove the cells from the surfaces by scraping, concentrate by centrifugation and demonstrate larger amount of DNase degradable DNA signal on adherent cells than on planktonic cells. Unfortunately, the number of adherent cells optimized for *in situ* counting was too low to provide a signal in ethidium bromide agarose gel electrophoresis. Also, ECM removed from scraped cells by suspending them in 1.5 M sodium chloride^42^ did not produce DNA signal on gel likely due to too low amount of DNA. PI + SYTO 9 staining and epifluorescence microscopy of 1.5 M NaCl-treated cells confirmed that most of the cells indeed stained green suggesting successful removal of ECM, including eDNA, from the cells. It was also empirically observed that physical manipulations of adherent cells from scraping to centrifugation, vortexing and ultrasonication all shifted the red to green staining ratio to more green, indicating (partial) ECM removal in various steps of the process which makes cell number normalization between planktonic and low numbers of adherent cells prior to analysis difficult to achieve without losing significant amounts of eDNA.

## Discussion

The need to study possible false dead results of PI-based viability staining arose from our previous experiments carried out with bacterial biofilms in water and PBS, where we have similarly to this study, observed a large fraction of red PI-stained cells in biofilms on untreated glass but significantly smaller fraction of red-stained cells on glass surfaces with antibacterial treatment, although total cell counts on treated surfaces tended to be much lower than on untreated controls (unpublished data; Supplementary Fig. 13; surfaces described in^43^). Yet the morphology of biofilms on untreated glass appeared normal while on antibacterial glass surfaces the biofilm structure as well as *S. epidermidis* characteristic diplococcal aggregation was disturbed. This result suggested almost reverse staining of alive and dead bacterial cells with PI and SYTO 9 in biofilms. In this study we show that in similar conditions on untreated glass membrane integrity based viability staining with NA-binding PI can and will significantly overestimate the dead cell count in 24 h gram-positive and gram-negative monospecies biofilms in PBS. 96.35 ± 5.30% of *E. coli* and 75.69 ± 18.44% of *S.epidermidis* cells stained red and according to general viability staining principles could be considered “dead” when co-staining with PI and SYTO 9 *in situ* compared to 67.91% *E. coli* and 68.30% *S. epidermidis* staining FDA-positive – metabolically active *in situ*, and at least 82.43% of *E. coli* and 89.02% of *S. epidermidis* cells being cultivable after harvesting from biofilms via ultrasonication. It was also evident that the red (PI) to green (SYTO 9) signal ratio was reversed after ultrasonication which indicates PI signal localization in the ECM and (partial) ECM removal during physical manipulation of the cells. To our knowledge, non-specific fluorescence of PI in biofilm ECM has not been described as a factor possibly influencing viability staining results. PI does also bind RNA, not only DNA. However, while the presence of eDNA in ECM is well described, not much is known about extracellular RNA (eRNA) in biofilms. It has been shown that bacteria can secrete RNA in outer membrane vesicles (OMVs). For example, Ghosal *et al*. have demonstrated eRNA outside planktonic E. coli MG1655^44^, the very same strain that was used in our experiments. Whether eRNA has a role in biofilm formation is yet scarcely studied, but it cannot be ruled out. For example, eRNA has been demonstrated to be important in *Haemophilus influenzae in vitro* biofilm formation^45^.

Compared to harvested cell plate count, FDA staining results *in situ* seem to underestimate viability (Table 1). This could be due to a few reasons. Firstly, the FDA method is challenging to work with due to weak fluorescent signals that require long exposures leading to photobleaching, high background fluorescence and varying signal intensities between individual cells (especially in the case of *S. epidermidis*). Secondly, FDA is indicative of metabolic activity, but biofilms were formed in a very nutrient-poor environment in which metabolic activity is expected to be slowed and as shown by Chavez de Paz *et al*. for oral bacteria, can reversibly affect FDA staining outcome^46^.

Confocal laser scanning microscopy revealed double-stained cells with green fluorescing interiors under red stained exteriors of individual cells and confirmed PI staining not only being indicative of membrane integrity but rather staining of eDNA which is one of the components of bacterial ECM. This double-staining was only characteristic of viable biofilms as ethanol-fixed biofilms consistently produced only red signals in both EM (Supplementary Figs 2 and 3) and CLSM (Supplementary Figs 8 and 9). Due to CLSM resolution limit, super-resolution microscopy allowing for nanoscale discrimination between membrane and chromosomal DNA signals^47^ would be needed to confidently confirm PI exclusion from cell interiors.

CLSM results look similar to what has been previously demonstrated, but not quantified in terms of falsely assigned dead signal counts for viability staining. For example, Vilain *et al*. demonstrated similar PI corona around adherent *Bacillus cereus* cells on glass wool^48^, although in their study, the biofilm was formed in rich medium. Gallo *et al*.^41^ also noted a similar picture using membrane-anchored surface amyloid fibril (SAF) producing and GFP-expressing *Salmonella typhimurium* biofilm cells surrounded by a “corona” of PI-stained eDNA and SAF complexes concluding that PI stains the cells externally. SAF and eDNA interactions have also been demonstrated for other species. For example, eDNA has been shown to facilitate the polymerization of SAF monomers in *Staphylococcus aureus* biofilms^49^ and *E. coli* SAF monomer has been shown to bind to DNA, promoting SAF assembly^50^. SAF and eDNA have been shown to facilitate bacterial attachment to surfaces and cell-cell aggregation^51^. SAF and eDNA interactions in the context of biofilm formation and mechanical resistance need to be studied further to bring light to underlying mechanisms. We also demonstrated that amyloid stain Congo red stained both *E. coli* and *S. epidermidis* (Supplementary Fig. 12). CR combined with SYTO 9 presented similar staining pattern in epifluorescence microscopy as PI + SYTO 9 leading to hypothesis that eDNA-SAF complexes could bind PI, mask intracellular SYTO 9 signals and lead to false dead signals in epifluorescence microscopy. In mammalian amyloid diseases research, it has been observed that amyloids not only bind DNA, but also mediate its configurational changes between B and Z form^52–54^. If it similarly applies to bacterial amyloids, then that might explain why this caveat in viability staining has not been shown to be a critical problem for older biofilms that quickly become insensitive to DNase during maturation^20^ and generally stain green with PI + SYTO 9. This could be due to Z-DNA not being efficiently degraded by DNase I^55^ nor detected by ethidium bromide (EB)^56^, latter of which is structurally very similar to PI.

SAF-bound eDNA could also explain why PI stains biofilms on untreated glass and not on nano-ZnO coated surfaces (Supplementary Fig. 13) that release ionic Zn(II). Zn(II) in known to modulate amyloid formation. Exact mechanisms and related interactions are not well known, but Zn(II) has been demonstrated to inhibit fibrillar growth or cause destabilization of amyloid fibrils^57–61^. If Zn(II) prevents functional SAF formation or disrupts existing amyloids, then it could also prevent SAF-bound eDNA in close proximity to the cells. More specifically, Tõugu *et al*.^57^ demonstrated dose-dependent inhibition of amyloid β fibril formation with strong effect at 5–10 µM Zn(II) in physiological conditions while Zn concentration in our biofilm experiment with nano-ZnO coated surfaces facilitating SYTO9-positive *S. epidermidis* biofilms in PBS reached about 15 µM with potentially higher local concentrations near the treated surface. The mechanisms behind DNA, amyloid and Zn or other metal ion interactions need further investigation and may reveal novel strategies to prevent biofilm formation.

Moreover, the role of eDNA in PI-staining of adherent bacterial cells may not be constant in different biofilms but significantly affected by biofilm growth conditions. In our study we used biofilms grown in nutrient-poor PBS at ambient temperature and no other conditions that could negatively affect adherent cultures were applied. However, in a more usual experimental setup, different treatments causing physical, toxic, starvation *etc*. stress to the biofilms, especially in antimicrobial or anti-biofilm research together with a negative no-stress control are used. In the light of eDNA interfering with viability staining results, these stress factors could not only affect cell viability but also adherence efficiency and with that the amount of ECM and eDNA thereby potentially falsely exaggerating mortality of stress-treated samples compared to no-stress controls. For example, metabolic stress due to sub-lethal concentrations of antibiotics or other toxic compounds has been shown to enhance biofilm formation and/or result in higher eDNA content of the biofilms^62–65^. Growth conditions, such as temperature, aerobic and starvation stress were reported to affect surface attachment and eDNA-mediated mechanism of biofilm formation of *Campylobacter jejuni*^66^. DNase-sensitive eDNA dependent biofilm formation of *Streptococcus mutans* was observed in low pH stress and not in neutral pH^67^. Higher eDNA content of biofilms subjected to physico-chemical stress was recently also observed for *S. epidermidis*^68^. Not only severe stress, but also growth media selection can be of importance. Kadam *et al*. observed highest biofilm formation in nutrient-poor mediums and noticed that DNase-sensitive *Listeria monocytogenes* biofilms grown in nutrient broth consisted of clearly higher proportion of PI-positive cells during PI + SYTO 9 co-staining than DNase-insensitive biofilms of the same strains grown in a more nutrient rich brain heart infusion^69^. Also SAF biogenesis, discussed above, is stimulated by temperature below 30 °C and lack of nutrients^70^.

Together, this hints that the external staining phenomenon of PI might not only be dependent on the species used or starvation conditions but is also attachment-specific and dependent on conditions affecting matrix eDNA content, including different stress-responses.

## Conclusion

Viability estimation is of critical importance in evaluating antimicrobial/anti-biofilm surfaces and substances efficiency. Although the presence of extracellular nucleic acids in bacterial biofilm matrixes is well established in the literature, PI-based viability staining has remained a widely used tool for *in situ* viability estimate of adherent cells not taking into account possible eDNA interference in the viability staining results. From this study it can be concluded that membrane integrity based viability staining with DNA-binding dyes, including, but presumably not limited by PI, can significantly overestimate dead cell counts in the presence of eNA in biofilms. To overcome this, the possible effect of eNA should be controlled for by either: (1) using culture-based methods as a reference; (2) assess metabolic activity (e.g. staining for enzyme activity, respiration etc.) in parallel to NA-staining and/or (3) minimizing ECM co-harvesting if harvested cell viability is to be assessed by staining. None of the aforementioned approaches are perfect for biofilms, but combination of methods rather than one approach is expected to result in more accurate estimations of viability.

## Methods

### Preparation of glass surfaces for bacterial attachment

18 mm × 18 mm soda-lime glass microscopy cover glasses (Corning, 2855-18) were used as biofilm carriers. Before inoculation carriers were rinsed with 70 vol% ethanol in deionized water and dried in biosafety cabinet with ultraviolet light irradiation for at least 20 minutes on both sides.

### Bacterial strains and biofilm cultivation

*S. epidermidis* DSM-20044 and *E. coli* MG1655 were grown overnight in Luria-Bertani broth (LB: 10 g/L tryptone, 5 g/L yeast extract in deionized water) at 30 °C. Sterilized 18 × 18 mm glass cover slips were placed into wells of 6-well polycarbonate non-tissue culture coated plates (Corning, 351146). Bacterial cells where washed twice with PBS (180 mM sodium chloride, 3 mM potassium chloride, 9 mM dibasic sodium phosphate, 1,5 mM potassium hydrogen phosphate in deionized water, pH~7) using centrifugation at 7000 g for 10 min. Cell suspensions were immediately diluted to OD_600_ 0.01 in PBS and 5 ml of inoculum was pipetted onto glass surfaces in each well of the 6-well plates. Serial dilutions of remaining inoculum were made and drop-plated on nutrient agar (NA: 5 g/L meat extract, 10 g/L peptone, 5 g/L sodium chloride, 15 g/L agar powder in deionized water) to confirm inoculum cell count. Plates with inoculated surfaces were covered with lids and incubated at room temperature and ambient indoor lighting for 24 h to acquire biofilm density suitable for consecutive counting.

Ethanol-fixation was used to kill and permeate biofilm samples used as controls. 24 h biofilms were dip-rinsed twice in PBS and drained. Biofilms were submerged in 70 vol% ethanol in deionized water, incubated 1 h at room temperature, liquid aspirated, and samples dried in 60 °C incubator for 5 min.

### Staining

Staining with PI (81845, Sigma) 20 mM and SYTO 9 (S-34854, Invitrogen^TM^ Thermo Fisher Scientific) 3.34 mM stock solutions in DMSO was carried out according to BacLight^TM^ Bacterial Viability Kit manual. Final concentrations of stains in 1:1 stain mixture in PBS was 30 µM PI and 5 µM SYTO 9. Stain mixture was either added to surfaces with biofilms (15 µl PBS-diluted stain mix pipetted straight onto surfaces and covered by cover slip), to cells harvested from surfaces by ultrasonication or to planktonic bacteria collected from above the bacterial biofilms. The stained samples were incubated for 15 minutes in the dark (foil covered box) at room temperature.

FDA (201642, Sigma) stock solution used was 5 mg/ml in acetone, diluted 200-fold in PBS and kept on ice during the experiment. 15 µl of the stain solution was pipetted directly onto surfaces, covered by coverslip and incubated in the dark for 10 min before microscopy. Longer incubation periods yielded in high background fluorescence and not significantly stronger signals from cells and therefore longer incubation was not used to obtain stronger signals.

Final concentrations for other stains combined with 5 µM SYTO 9: 20 µg/ml Congo red (Merck), 1 µg/ml Nile red (Thermo Fisher Scientific), 5 µg/ml (1×) CellMask Orange (Thermo Fisher Scientific). Same staining conditions were applied as for PI + SYTO 9, described above.

For membrane potential evaluation, 15 µl 30 µM 3,3′-diethyloxacarbocyanine iodide (DiOC_2_(3); 320684, Sigma) in PBS was used *in situ* followed by 5 min incubation in the dark and epifluorescence microscopy with or without 5 min pre-treatment submerged in 5 µM carbonyl cyanide m-chlorophenylhydrazone (CCCP; C2759, Sigma) in PBS. DiOC_2_(3) used by Kirchhoff and Cypionka^5^ was chosen over Thioflavin T used by Humphries *et al*.^37^ for membrane potential staining due to Thioflavin T being a widely used amyloid dye and potentially staining also amyloid fibrils^57^ present in biofilm matrix.

### Ultrasonication

Branson Digital Sonifier model 450 (max power 400 W) equipped with horn model 101-135-066 R was used to harvest adherent cells from glass surfaces. The protocol was optimized to achieve maximal viable cell yield for ultrasonication of glass surfaces in 50 ml glass beaker filled with 10 ml PBS at 25% sonication amplitude. For optimization, planktonic culture was used in parallel to biofilm and viability of both planktonic and harvested cells was evaluated during up to 30 sec sonication (Supplementary Fig. 6). Optimal time for sonication to achieve maximal viable cell yield was found to be 15 seconds for both bacterial species. Ultrasonicated surfaces were stained with PI and SYTO 9 and microscoped to confirm removal of biofilm. Harvested biofilm samples were either stained as described above and analyzed by flow cytometry or filtered through 0.2 µm pore size filters (Whatman Nuclepore Polycarbonate Black Membrane Filter) prior to microscopy and counting or drop-plated for CFU counts on nutrient agar. For more reproducible result presentation, CFU and cell counts are given per cm^2^.

### Microscopy

Epifluorescence microscopy (EM) was carried out using Olympus CX41 microscope equipped with 100x oil immersion objective. Excitation filter cube DMB-2 (exciter filter BP475, dichroic mirror DM500, barrier filter O515IF) was used to filter mercury lamp emission allowing detection of both FDA as well as simultaneous detection of PI and SYTO 9 fluorescent signals with 515 nm longpass filter. Images were captured with Olympus DP71 camera and Cell^B software.

Signals were manually counted in ImageJ software using “point” tool thereby acquiring cell counts for *E. coli* and diplococcal counts for *S. epidermidis*. Compact diplococci with one green and one red cell were counted as separate signals. For counting purposes at least 10 images were taken per sample at random locations. “Subtract background” (rolling = 50) function of ImageJ was used on FDA-stained images prior to counting signals with recognizable cell morphology. For more reproducible result presentation, cell/diplococcal counts are given per cm^2^.

Confocal laser scanning microscopy (CSLM) was carried out using Zeiss LSM 510 META equipped with 100x oil immersion objective and acquired images analyzed in Zeiss LSM Image Browser. For acquiring SYTO 9 signals, 488 nm laser and 505–550 nm emission filter was used. For PI, NL and CMO, 561 nm laser in combination with 575 nm longpass emission filter was used. Separate tracks were employed for both excitation/emission paths to avoid signal bleed-through between emission channels. To obtain more precise Z-stack imaging interval, motorized piezo stage was used to image at 0.1 µm interval (PI + SYTO9 samples) or at 0.15 µm (SYTO 9 + NL/CMO). Full Z-stacks for cross-sections on Figs 3 and 4 can be found in Supplementary Album 1.

Full width at half maximum (FWHM) was measured from non-saturated double-stained cells separately from green and red channels from the same line selection in ImageJ using Gaussian fit function.

### Flow cytometry

FCM analysis of PI and SYTO 9 co-stained bacteria was carried out using BD Accuri™ C6 device (BD Biosciences). Primary forward scatter (FCS-H) and secondary fluorescence signal (FL1-H) thresholds were used to filter out noise with minimal loss in bacterial cell signals and live-dead gating was done for *E. coli* and *S. epidermidis* using different proportions of viable overnight culture and ethanol-killed overnight culture (1 h incubation in 70% ethanol) confirmed by plate counts. Gating of dead and alive signal populations was executed on SYTO 9 (FL1-A; 533/30 nm)/Propidium iodide (FL3-A; 670 nm LP) scatter plot as illustrated on Supplementary Fig. 7. For more reproducible result presentation, event counts are given per cm^2^.

### DNase treatment

15 surfaces per condition were prepared and rinsed as described and incubated with 500 µl 1x DNase I buffer (10x buffer: 100 mM Tris-HCl (pH 7.5), 25 mM MgCl_2_, 1 mM CaCl_2_) with or without DNase I (final concentration 100 U/ml, EN0523, Thermo Fisher Scientific). As a planktonic control, 3 ml of *E. coli* and 20 ml of *S. epidermidis* planktonic fraction, with estimated cell count similar to adherent cells on 15 surfaces were pelleted at 7000 g for 10 minutes, supernatant discarded and pellet suspended in DNase buffer with or without DNase I. Both, surfaces with biofilm and tubes with planktonic bacteria were incubated at 37 °C for 4 hours. Adherent cells were harvested by scraping with cell scraper in the same buffer and pelleted by centrifugation at 7000 g for 10 min, suspended in 300 µl 1.5 M NaCl to remove ECM as described in^42^, thoroughly vortexed and pelleted again to remove cells from ECM fraction. 30 µl of ECM fraction in the supernatant was run on agarose gel electrophoresis (0.8% agarose in Tris-acetate-EDTA (TAE) buffer, stained with 0.5 μg/ml ethidium bromide; 60 V, 60 min, visualized on UV-transilluminator). Pelleted cells were resuspended in PI and SYTO 9 co-stain solution in final concentrations as described above and either analyzed by FCM or 5 µl pipetted onto microscopy slide, covered by cover slip, incubated in dark for 15 min and visualized with epifluorescence microscope.

### Statistical analysis

Mean values and standard deviations were calculated by Microsoft Excel standard functions. P-values used in Fig. 2 were acquired using analysis of variance (ANOVA) followed by Tuckey’s multiple comparisons test at α = 0.05 in GraphPadPrism 7.04 where analysis was executed individually for data presented on each graph (Fig. 2a–d). P-values used in Supplementary Fig. 10 were calculated in Microsoft Excel using two-tailed T-test.

## Supplementary information

Supplementary Figures

Supplementary Album 1

## Data Availability

The data generated in the current study is available from the corresponding author on reasonable request.

## References

[1] Boulos L, Prévost M, Barbeau B, Coallier J, Desjardins R (1999). LIVE/DEAD® BacLight^TM^: application of a new rapid staining method for direct enumeration of viable and total bacteria in drinking water. J. Microbiol. Methods.

[2] Stocks SM (2004). Mechanism and use of the commercially available viability stain,BacLight. Cytometry.

[3] User Manual: LIVE/DEAD BacLight Bacterial Viability Kits. (2004).

[4] Stiefel P, Schmidt-Emrich S, Maniura-Weber K, Ren Q (2015). Critical aspects of using bacterial cell viability assays with the fluorophores SYTO9 and propidium iodide. BMC Microbiol..

[5] Kirchhoff C, Cypionka H (2017). Propidium ion enters viable cells with high membrane potential during live-dead staining. J. Microbiol. Methods.

[6] Yang, Y., Xiang, Y. & Xu, M. From red to green: the propidium iodide-permeable membrane of Shewanella decolorationis S12 is repairable. *Sci. Rep*. **5** (2016). 10.1038/srep18583 PMC4685271 26687136

[7] Gião MS, Wilks SA, Azevedo NF, Vieira MJ, Keevil CW (2009). Validation of SYTO 9/Propidium Iodide Uptake for Rapid Detection of Viable but Noncultivable Legionella pneumophila. Microb. Ecol..

[8] Auty MAE (2001). Direct *In Situ* Viability Assessment of Bacteria in Probiotic Dairy Products Using Viability Staining in Conjunction with Confocal Scanning Laser Microscopy. Appl. Environ. Microbiol..

[9] Zhu M, Takenaka S, Sato M, Hoshino E (2001). Influence of starvation and biofilm formation on acid resistance of Streptococcus mutans. Oral Microbiol. Immunol..

[10] Guggenheim B, Giertsen E, Schüpbach P, Shapiro S (2001). Validation of an *in vitro* Biofilm Model of Supragingival Plaque. J. Dent. Res..

[11] Azeredo J (2017). Critical review on biofilm methods. Crit. Rev. Microbiol..

[12] Magana M (2018). Options and Limitations in Clinical Investigation of Bacterial Biofilms. Clin. Microbiol. Rev..

[13] Rice KC (2007). The cidA murein hydrolase regulator contributes to DNA release and biofilm development in Staphylococcus aureus. Proc. Natl. Acad. Sci..

[14] Haagensen JAJ (2007). Differentiation and Distribution of Colistin- and Sodium Dodecyl Sulfate-Tolerant Cells in Pseudomonas aeruginosa Biofilms. J. Bacteriol..

[15] Hope CK, Clements D, Wilson M (2002). Determining the spatial distribution of viable and nonviable bacteria in hydrated microcosm dental plaques by viability profiling. J. Appl. Microbiol..

[16] Webb JS (2003). Cell Death in Pseudomonas aeruginosa Biofilm Development. J. Bacteriol..

[17] Guilbaud M, Piveteau P, Desvaux M, Brisse S, Briandet R (2015). Exploring the Diversity of Listeria monocytogenes Biofilm Architecture by High-Throughput Confocal Laser Scanning Microscopy and the Predominance of the Honeycomb-Like Morphotype. Appl. Environ. Microbiol..

[18] Shi L (2007). Limits of propidium iodide as a cell viability indicator for environmental bacteria. Cytometry A.

[19] Whitchurch CB (2002). Extracellular DNA Required for Bacterial Biofilm Formation. Science.

[20] Okshevsky M, Meyer RL (2015). The role of extracellular DNA in the establishment, maintenance and perpetuation of bacterial biofilms. Crit. Rev. Microbiol..

[21] Hymes SR, Randis TM, Sun TY, Ratner AJ (2013). DNase Inhibits Gardnerella vaginalis Biofilms *In Vitro* and *In Vivo*. J. Infect. Dis..

[22] Okshevsky M, Regina VR, Meyer RL (2015). Extracellular DNA as a target for biofilm control. Curr. Opin. Biotechnol..

[23] Àlvarez G, González M, Isabal S, Blanc V, León R (2013). Method to quantify live and dead cells in multi-species oral biofilm by real-time PCR with propidium monoazide. AMB Express.

[24] Nocker A, Camper AK (2009). Novel approaches toward preferential detection of viable cells using nucleic acid amplification techniques. FEMS Microbiol. Lett..

[25] Reyneke B, Ndlovu T, Khan S, Khan W (2017). Comparison of EMA-, PMA- and DNase qPCR for the determination of microbial cell viability. Appl. Microbiol. Biotechnol..

[26] Okshevsky M, Meyer RL (2014). Evaluation of fluorescent stains for visualizing extracellular DNA in biofilms. J. Microbiol. Methods.

[27] Allesen-Holm M (2006). A characterization of DNA release in Pseudomonas aeruginosa cultures and biofilms. Mol. Microbiol..

[28] Mann EE (2009). Modulation of eDNA Release and Degradation Affects Staphylococcus aureus Biofilm Maturation. PLoS ONE.

[29] Gião MS, Keevil CW (2014). Listeria monocytogenes Can Form Biofilms in Tap Water and Enter Into the Viable but Non-Cultivable State. Microb. Ecol..

[30] Ito A, May T, Kawata K, Okabe S (2008). Significance of *rpoS* during maturation of *Escherichia coli* biofilms. Biotechnol. Bioeng..

[31] Wood TK, González Barrios AF, Herzberg M, Lee J (2006). Motility influences biofilm architecture in Escherichia coli. Appl. Microbiol. Biotechnol..

[32] Gonzalez Barrios AF (2006). Autoinducer 2 Controls Biofilm Formation in Escherichia coli through a Novel Motility Quorum-Sensing Regulator (MqsR, B3022). J. Bacteriol..

[33] Bayramoglu, B., Toubiana, D. & Gillor, O. Genome-wide transcription profiling of aerobic and anaerobic *Escherichia coli* biofilm and planktonic cultures. *FEMS Microbiol. Lett*. fnx006, 10.1093/femsle/fnx006 (2017). 10.1093/femsle/fnx006 28087619

[34] Lacqua A, Wanner O, Colangelo T, Martinotti MG, Landini P (2006). Emergence of Biofilm-Forming Subpopulations upon Exposure of *Escherichia coli* to Environmental Bacteriophages. Appl. Environ. Microbiol..

[35] Qin Z (2007). Role of autolysin-mediated DNA release in biofilm formation of Staphylococcus epidermidis. Microbiology.

[36] Wang R (2012). Inhibition of escherichia coli and proteus mirabilis adhesion and biofilm formation on medical grade silicone surface. Biotechnol. Bioeng..

[37] Humphries J (2017). Species-Independent Attraction to Biofilms through Electrical Signaling. Cell.

[38] Lundgren B (1981). Fluorescein Diacetate as a Stain of Metabolically Active Bacteria in Soil. Oikos.

[39] Comte S, Guibaud G, Baudu M (2006). Relations between extraction protocols for activated sludge extracellular polymeric substances (EPS) and EPS complexation properties. Enzyme Microb. Technol..

[40] Pan, X. *et al*. A comparison of five extraction methods for extracellular polymeric substances (EPS) from biofilm by using threedimensional excitation-emission matrix (3DEEM) fluorescence spectroscopy. *Water SA* **36** (2010).

[41] Gallo PM (2015). Amyloid-DNA Composites of Bacterial Biofilms Stimulate Autoimmunity. Immunity.

[42] Chiba A, Sugimoto S, Sato F, Hori S, Mizunoe Y (2015). A refined technique for extraction of extracellular matrices from bacterial biofilms and its applicability: Extraction of ECM from bacterial biofilms. Microb. Biotechnol..

[43] Visnapuu M (2018). UVA-induced antimicrobial activity of ZnO/Ag nanocomposite covered surfaces. Colloids Surf. B Biointerfaces.

[44] Ghosal A (2015). The extracellular RNA complement of *Escherichia coli*. MicrobiologyOpen.

[45] Domenech, M., Pedrero-Vega, E., Prieto, A. & García, E. Evidence of the presence of nucleic acids and β-glucan in the matrix of non-typeable Haemophilus influenzae *in vitro* biofilms. *Sci. Rep*. **6** (2016). 10.1038/srep36424 PMC5090351 27805043

[46] Chavez de Paz LE, Hamilton IR, Svensater G (2008). Oral bacteria in biofilms exhibit slow reactivation from nutrient deprivation. Microbiology.

[47] Spahn, C. K. *et al*. A toolbox for multiplexed super-resolution imaging of the *E. coli* nucleoid and membrane using novel PAINT labels. *Sci. Rep*. **8** (2018). 10.1038/s41598-018-33052-3 PMC6170473 30282984

[48] Vilain S, Pretorius JM, Theron J, Brozel VS (2009). DNA as an Adhesin: Bacillus cereus Requires Extracellular DNA To Form Biofilms. Appl. Environ. Microbiol..

[49] Schwartz K, Ganesan M, Payne DE, Solomon MJ, Boles BR (2016). Extracellular DNA facilitates the formation of functional amyloids in *S taphylococcus aureus* biofilms: eDNA promotes functional amyloid formation. Mol. Microbiol..

[50] Fernández-Tresguerres ME, Moreno-Díaz de la Espina S, Gasset-Rosa F, Giraldo RA (2010). DNA-promoted amyloid proteinopathy in *Escherichia coli*: Synthetic bacterial amyloidosis. Mol. Microbiol..

[51] Van Gerven N, Van der Verren SE, Reiter DM, Remaut H (2018). The Role of Functional Amyloids in Bacterial Virulence. J. Mol. Biol..

[52] Suram A, Rao JKS, Latha KS, Viswamitra MA (2002). First Evidence to Show the Topological Change of DNA from B-DNA to Z-DNA Conformation in the Hippocampus of Alzheimer’s Brain. NeuroMolecular Med..

[53] Yu H, Ren J, Qu X (2007). Time-Dependent DNA Condensation Induced by Amyloid β-Peptide. Biophys. J..

[54] Hegde ML (2004). First Evidence for Helical Transitions in Supercoiled DNA by Amyloid β Peptide (1–42) and Aluminum: A New Insight in Understanding Alzheimer’s Disease. J. Mol. Neurosci..

[55] Suck D, Oefner C (1986). Structure of DNase I at 2.0 Å resolution suggests a mechanism for binding to and cutting DNA. Nature.

[56] Walker GT, Stone MP, Krugh TR (1985). Ethidium binding to left-handed (Z) DNAs results in regions of right-handed DNA at the intercalation site. Biochemistry.

[57] Tõugu V (2009). Zn(II)- and Cu(II)-induced non-fibrillar aggregates of amyloid-β (1–42) peptide are transformed to amyloid fibrils, both spontaneously and under the influence of metal chelators. J. Neurochem..

[58] Sarell CJ, Wilkinson SR, Viles JH (2010). Substoichiometric Levels of Cu^2+^ Ions Accelerate the Kinetics of Fiber Formation and Promote Cell Toxicity of Amyloid-β from Alzheimer Disease. J. Biol. Chem..

[59] Abelein, A., Gräslund, A. & Danielsson, J. Zinc as chaperone-mimicking agent for retardation of amyloid β peptide fibril formation. *Proc. Natl. Acad. Sci*. **112**, 5407–5412 (2015). 10.1073/pnas.1421961112 PMC4418866 25825723

[60] Ma B, Zhang F, Wang X, Zhu X (2017). Investigating the inhibitory effects of zinc ions on amyloid fibril formation of hen egg-white lysozyme. Int. J. Biol. Macromol..

[61] Ban DK, Paul S (2016). Nano Zinc Oxide Inhibits Fibrillar Growth and Suppresses Cellular Toxicity of Lysozyme Amyloid. ACS Appl. Mater. Interfaces.

[62] Chang W (2015). Methicillin-Resistant Staphylococcus aureus Grown on Vancomycin-Supplemented Screening Agar Displays Enhanced Biofilm Formation. Antimicrob. Agents Chemother..

[63] Marchal M (2011). Subinhibitory Arsenite Concentrations Lead to Population Dispersal in Thiomonas sp. PLoS ONE.

[64] Doroshenko N (2014). Extracellular DNA Impedes the Transport of Vancomycin in Staphylococcus epidermidis Biofilms Preexposed to Subinhibitory Concentrations of Vancomycin. Antimicrob. Agents Chemother..

[65] Schilcher K (2016). Modulation of Staphylococcus aureus Biofilm Matrix by Subinhibitory Concentrations of Clindamycin. Antimicrob. Agents Chemother..

[66] Feng, J., Ma, L., Nie, J., Konkel, M. E. & Lu, X. Environmental Stress-Induced Bacterial Lysis and Extracellular DNA Release Contribute to *Campylobacter jejuni* Biofilm Formation. *Appl. Environ. Microbiol*. **84** (2017). 10.1128/AEM.02068-17 PMC5812928 29269493

[67] Kawarai T, Narisawa N, Suzuki Y, Nagasawa R, Senpuku H (2016). Streptococcus mutans biofilm formation is dependent on extracellular DNA in primary low pH conditions. J. Oral Biosci..

[68] Olwal, C. O., Ang’ienda, P. O., Onyango, D. M. & Ochiel, D. O. Susceptibility patterns and the role of extracellular DNA in Staphylococcus epidermidis biofilm resistance to physico-chemical stress exposure. *BMC Microbiol*. **18** (2018). 10.1186/s12866-018-1183-y PMC5930741 29720089

[69] Kadam SR (2013). Diversity assessment of Listeria monocytogenes biofilm formation: Impact of growth condition, serotype and strain origin. Int. J. Food Microbiol..

[70] Barnhart MM, Chapman MR (2006). Curli Biogenesis and Function. Annu. Rev. Microbiol..

