# Supplementary material for: Propidium iodide staining underestimates viability of adherent bacterial cells
Source: Sci Rep. 2019 Apr 24;9:6483. doi: 10.1038/s41598-019-42906-3 (PMC6482146; doi:10.1038/s41598-019-42906-3)
Supplement: Supplementary file 1 — Supplementary Figures [file 41598_2019_42906_MOESM1_ESM.pdf]

Propidium iodide staining underestimates viability of adherent bacterial cells

Merilin Rosenberg* [1, 2], Nuno F. Azevedo[3], Angela Ivask[1]

[1] Laboratory of Environmental Toxicology; National Institute of Chemical Physics and Biophysics; Akadeemia tee 23, 12618 Tallinn, Estonia
[2] Department of Chemistry and Biotechnology; Tallinn University of Technology; Akadeemia tee 15, 12618 Tallinn, Estonia
[3] LEPABE - Laboratory for Process Engineering, Environment, Biotechnology and Energy; Department of Chemical Engineering; Faculty of Engineering; University of Porto; Rua Dr. Roberto Frias, 4200-465 Porto, Portugal

* rosenbergmerilin@gmail.com; (+372) 5268 384

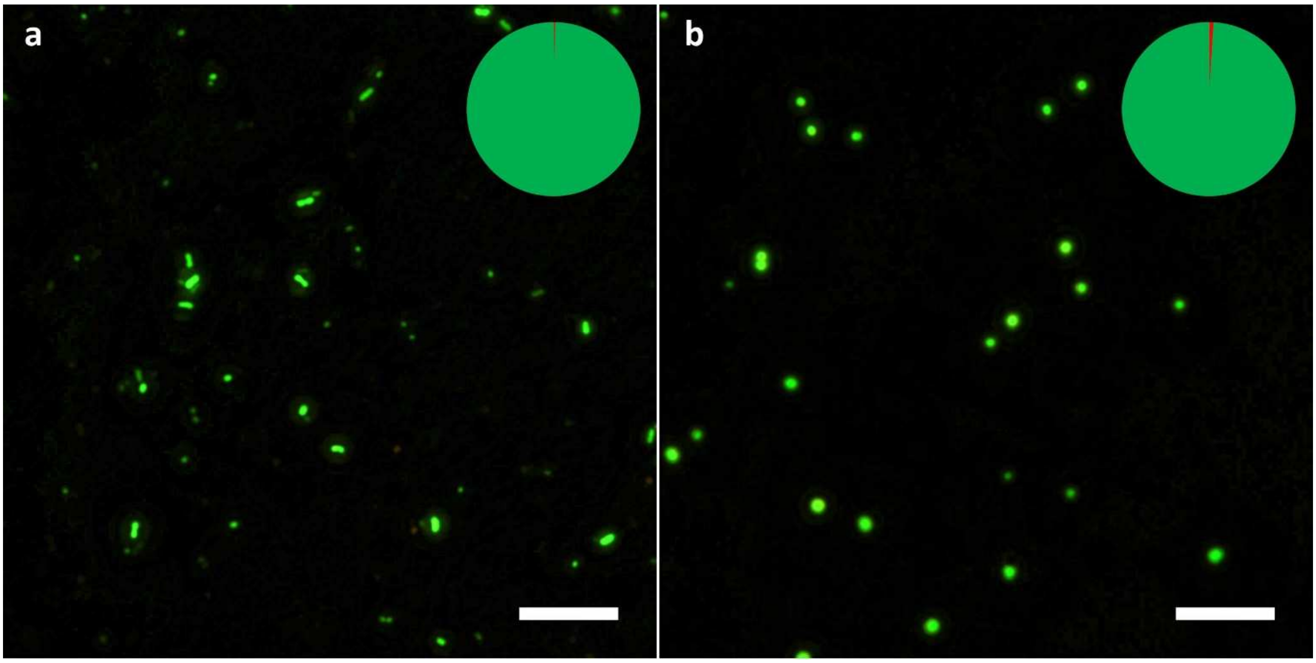

Supplementary Figure 1
Epifluorescence microscopy images of planktonic *E. coli* (a) and S. *epidermidis* (b) viability staining: planktonic cells from the biofilm experiment after 24 h incubation in phosphate buffered saline (PBS), stained with PI and SYTO 9 and collected on filter. Pie diagrams represent total cell count with PI, and SYTO 9 stained signal proportions marked in red and dark green respectively. Scale bars correspond to 10 µm.

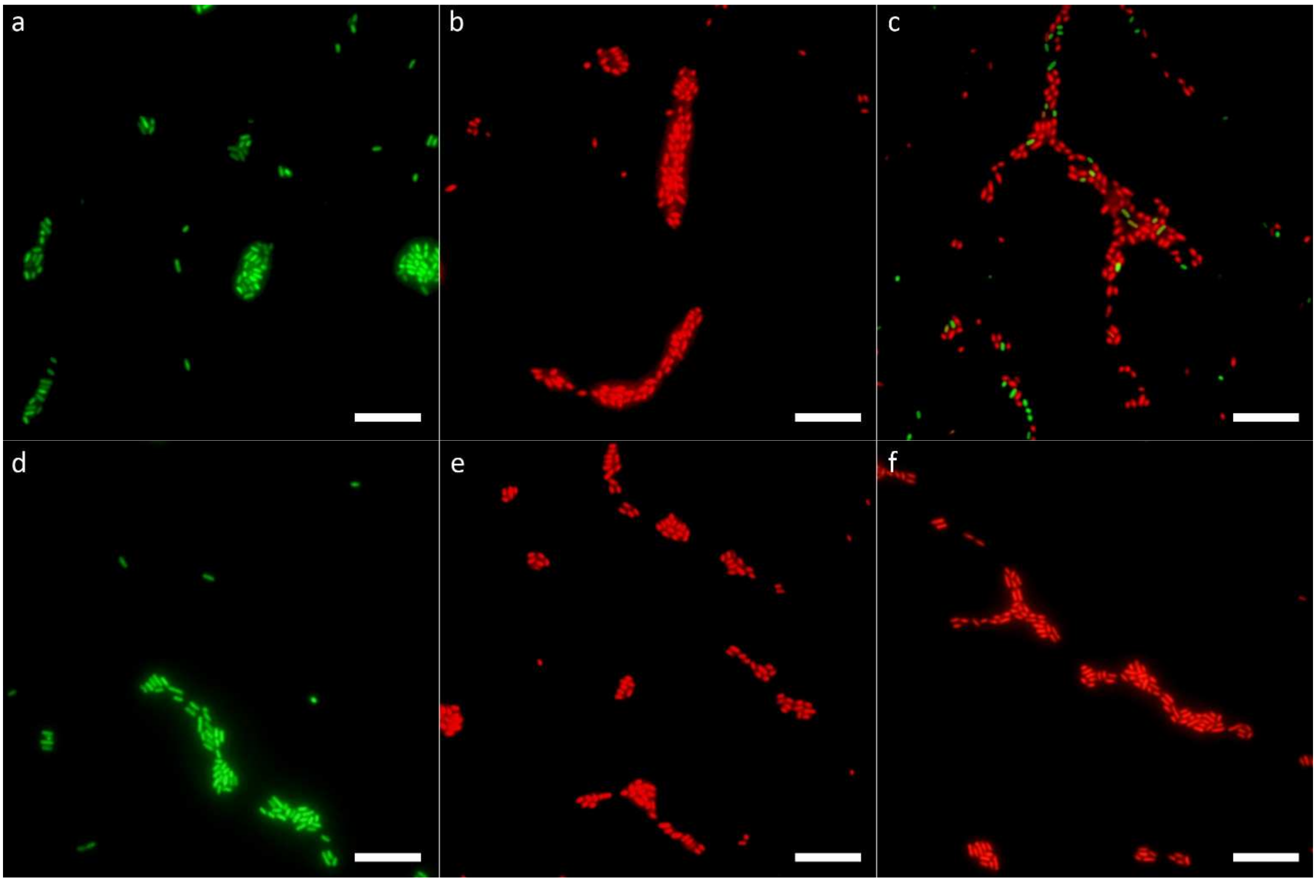

Supplementary Figure 2

Epifluorescence microscopy images of viable (a-c) and ethanol-fixed (d-e) 24 h *E. coli* biofilms stained with SYTO 9 (a, d), PI (b, e) or PI + SYTO 9 (c, f). Scale bars correspond to 10 µm.

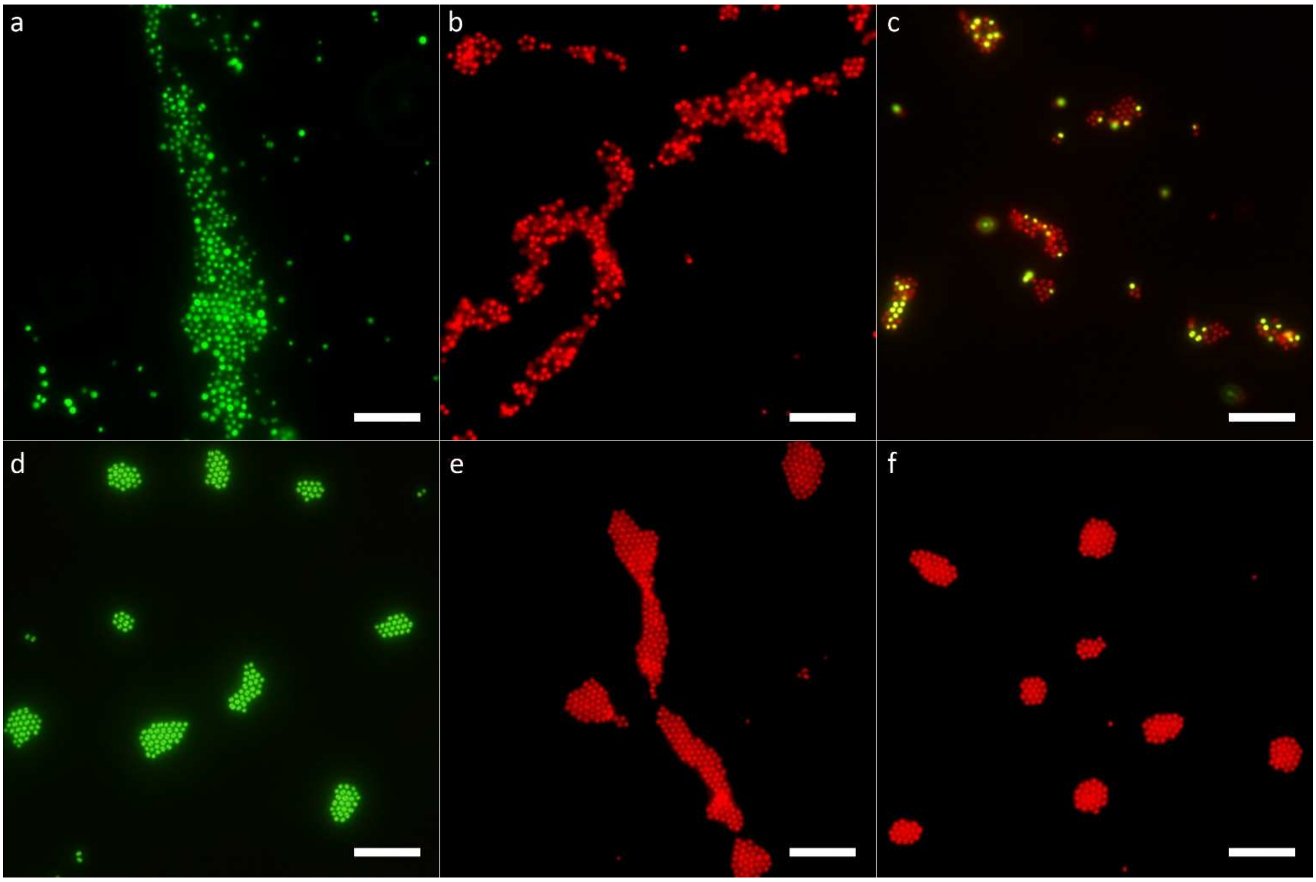

Supplementary Figure 3
Epifluorescence microscopy images of viable (a-c) and ethanol-fixed (d-e) 24 h *S. epidermidis* biofilms stained with SYTO 9 (a, d), PI (b, e) or PI + SYTO 9 (c, f). Scale bars correspond to 10 µm.

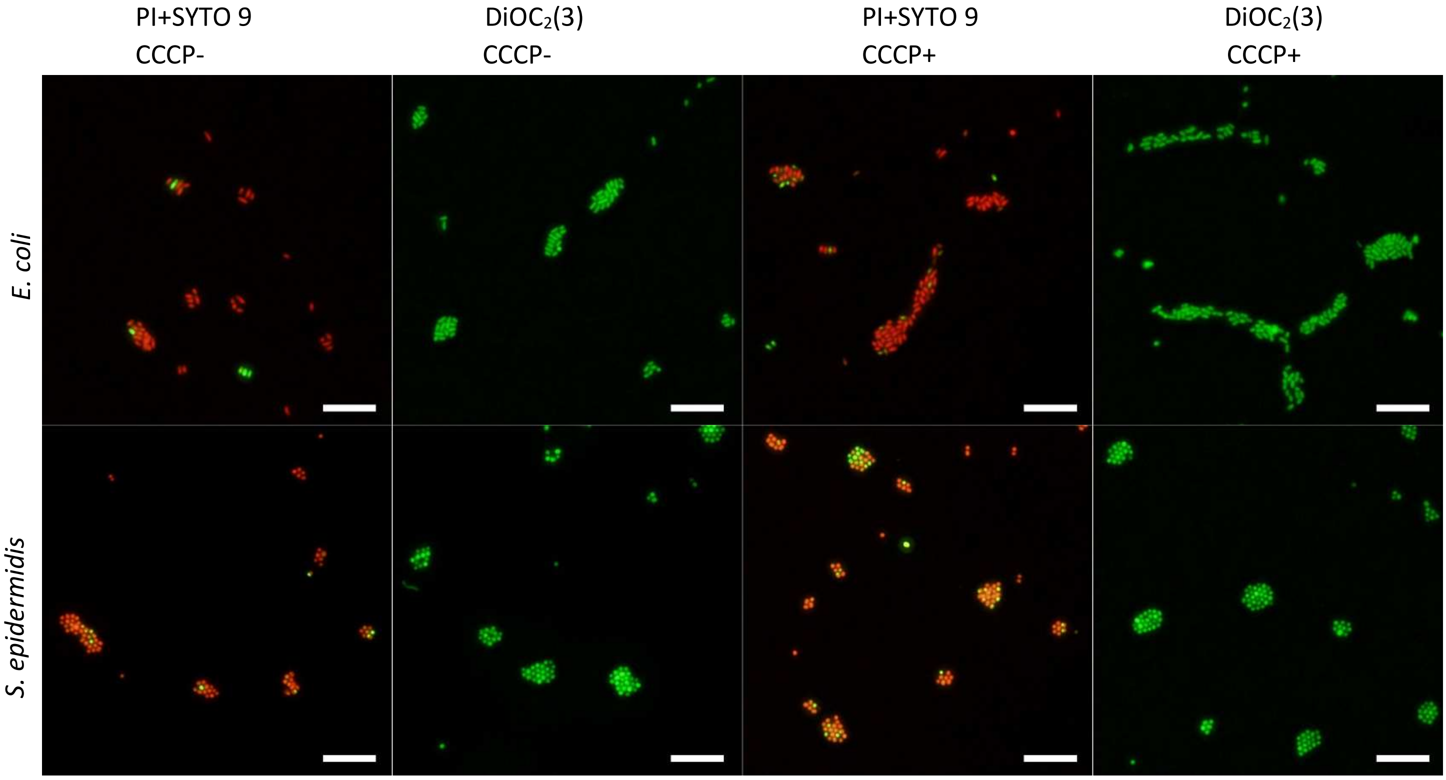

Supplementary Figure 4

Representative epifluorescence microscopy images of 24 h biofilms of *E. coli* and *S. epidermidis* stained in situ with PI + SYTO 9 or with $DiOC_2(3)$ with or without membrane potential relieving CCCP pre-treatment. No increased membrane potential signals were observed without CCCP pretreatment (CCCP-) and CCCP pretreatment (CCCP+) did not change overall PI + SYTO 9 staining pattern. Therefore, PI + SYTO 9 staining pattern is not caused by increased membrane potential of adherent cells. Scale bars correspond to 10 µm.

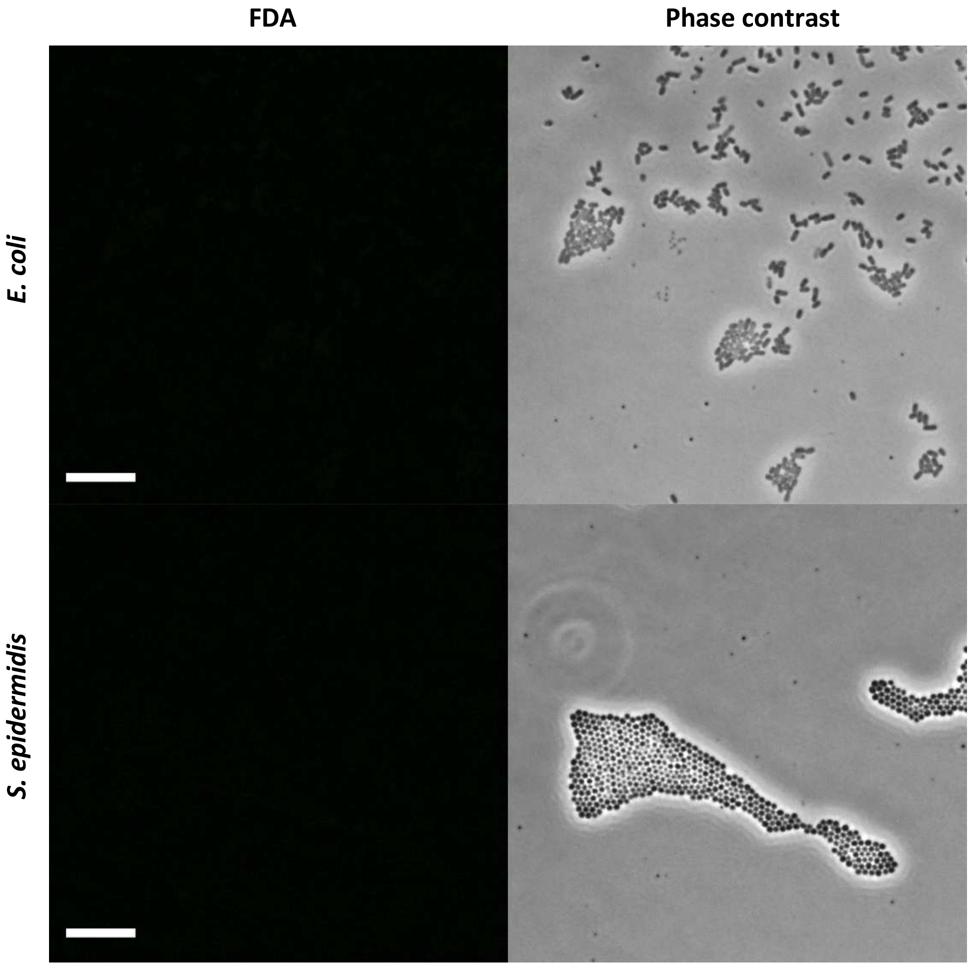

Supplementary Figure 5 Epifluorescence and phase contrast images of ethanol-fixed *E. coli* and *S. epidermidis* stained with fluorescein diacetate (FDA). Fluorescence and phase contrast image are captured from the same view field. No background FDA signals from killed cells are captured. Scale bars correspond to 10 µm.

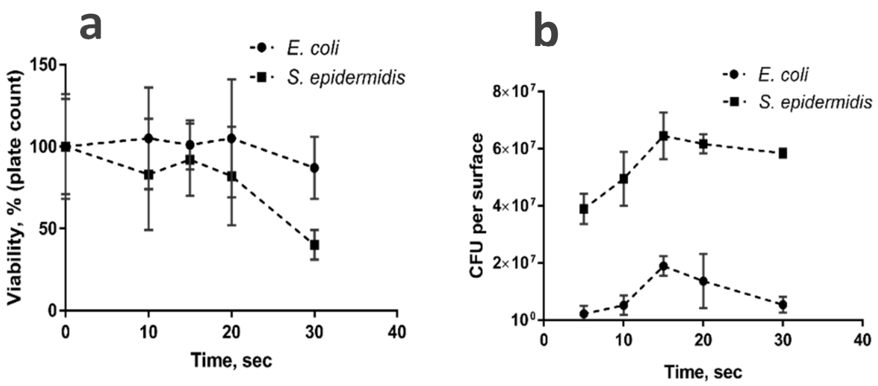

Supplementary Figure 6

Optimization of protocol for ultrasonication of adherent bacteria from biofilms on glass surfaces. Viability of planktonic bacteria (OD_{600}=0.05 in 10 ml PBS) during 0, 10, 15, 20 and 30 seconds sonication at 25% amplitude (a) and release of viable cells/aggregates from 24 h biofilm (OD_{600}=0.05 inoculum) in 10 ml PBS under the same conditions (b). Although planktonic culture endures longer treatment without decreasing viability, 15 seconds ultrasonication resulted in highest CFU per surface yield for biofilm cells. Glass surfaces were stained with propidium iodide and SYTO 9 after ultrasonication to confirm removal of biofilm.

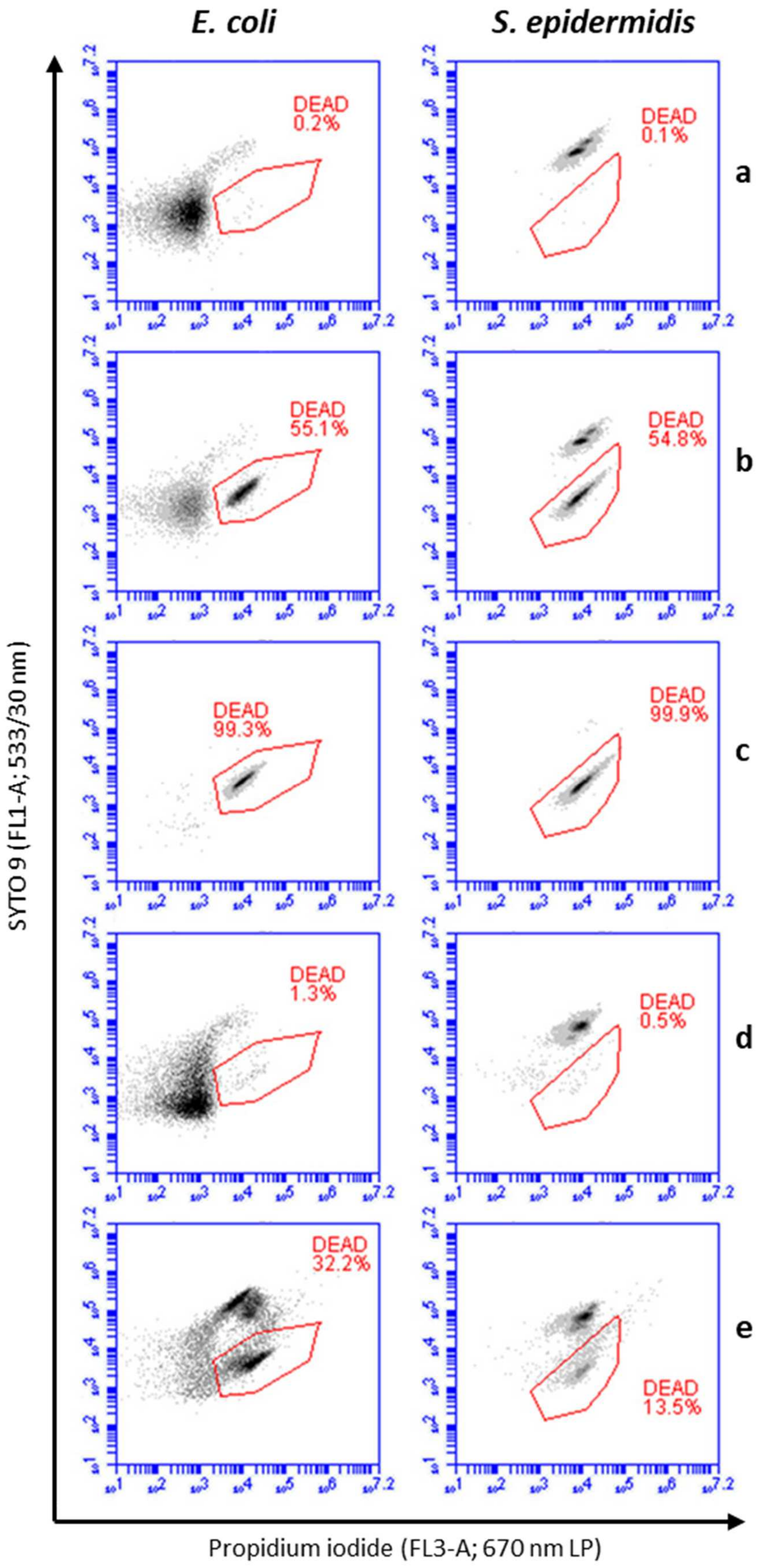

Supplementary Figure 7
Flow cytometry (FCM) density plots of propidium iodide (PI) and SYTO 9 co-stained *E.coli* MG1655 and *S. epidermidis* DSM20044: PBS washed overnight planktonic culture (a); 1:1 mix of viable and ethanol-killed overnight cultures (b); PBS washed and ethanol-killed overnight culture (c); pooled and ultrasonicated planktonic cells from biofilm experiment (d); ultrasonicated and pooled sessile cells from biofilm (e). Live/dead gating based on known proportions of viable and ethanol-killed planktonic bacteria was used to evaluate viability of bacteria harvested from 24 h biofilms. It is evident that gating strategy used is applicable for planktonic bacteria from the biofilm experiment even after incubating in PBS and sonication (d) while FCM populations of harvested *E. coli* biofilm cells have shifted and plots are much noisier. For adherent cells, dead gate seems to be better defined by PI staining than shifting populations of viable cells with presumably variable degrees of extracellular PI staining.

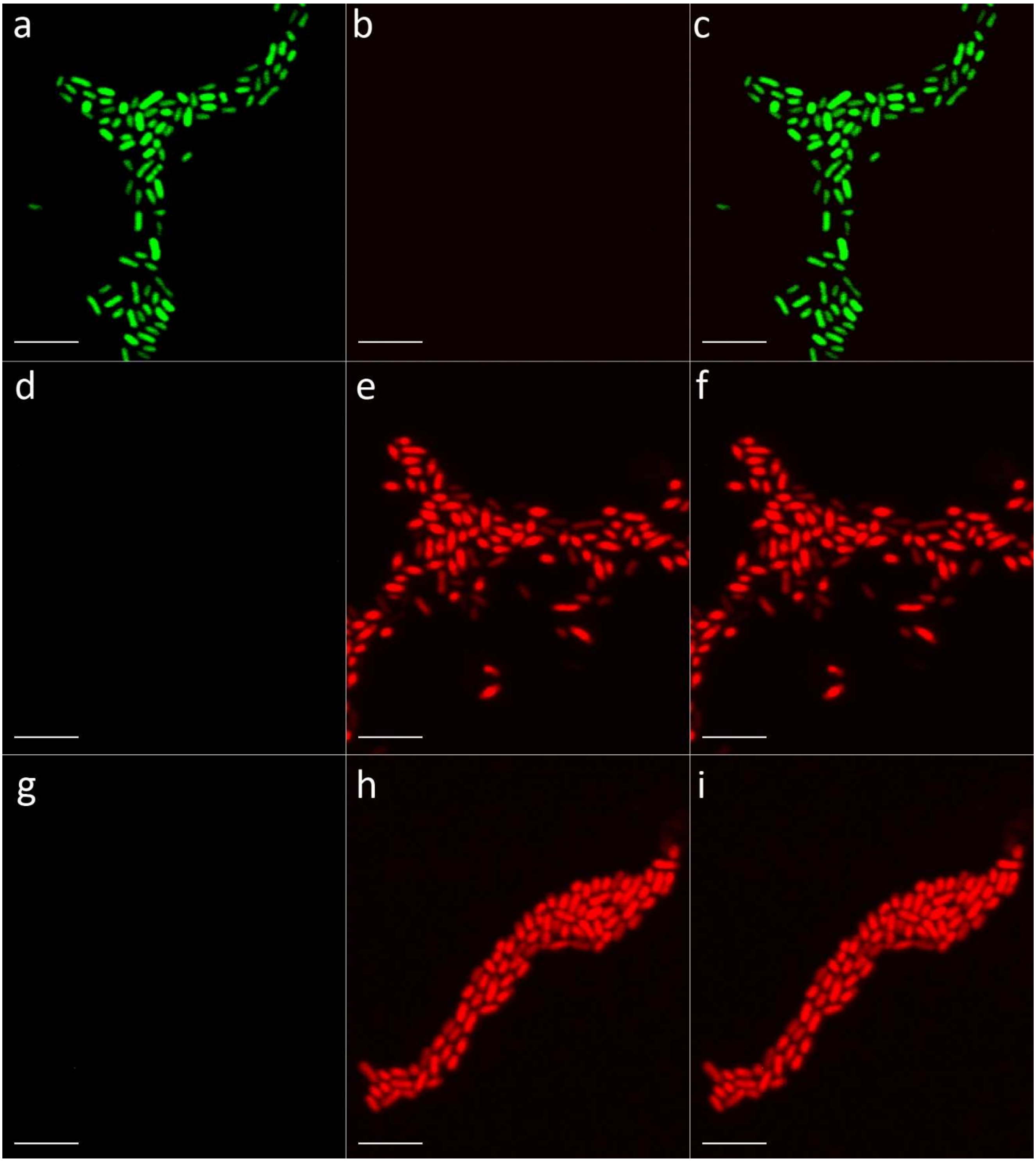

Supplementary Figure 8
Maximum projections of CLSM Z-stacks of viable *E. coli* biofilm stained with SYTO 9 (a-c) or PI (d-f) and **ethanol-fixed biofilm** stained with PI + SYTO 9 (g-i). Green channel (a, d, g), red channel, (b, e, h) and multichannel (c, f, i) projections are presented. The same acquisition and linear post-adjustment settings were applied to all images. Scale bars correspond to 5 µm.

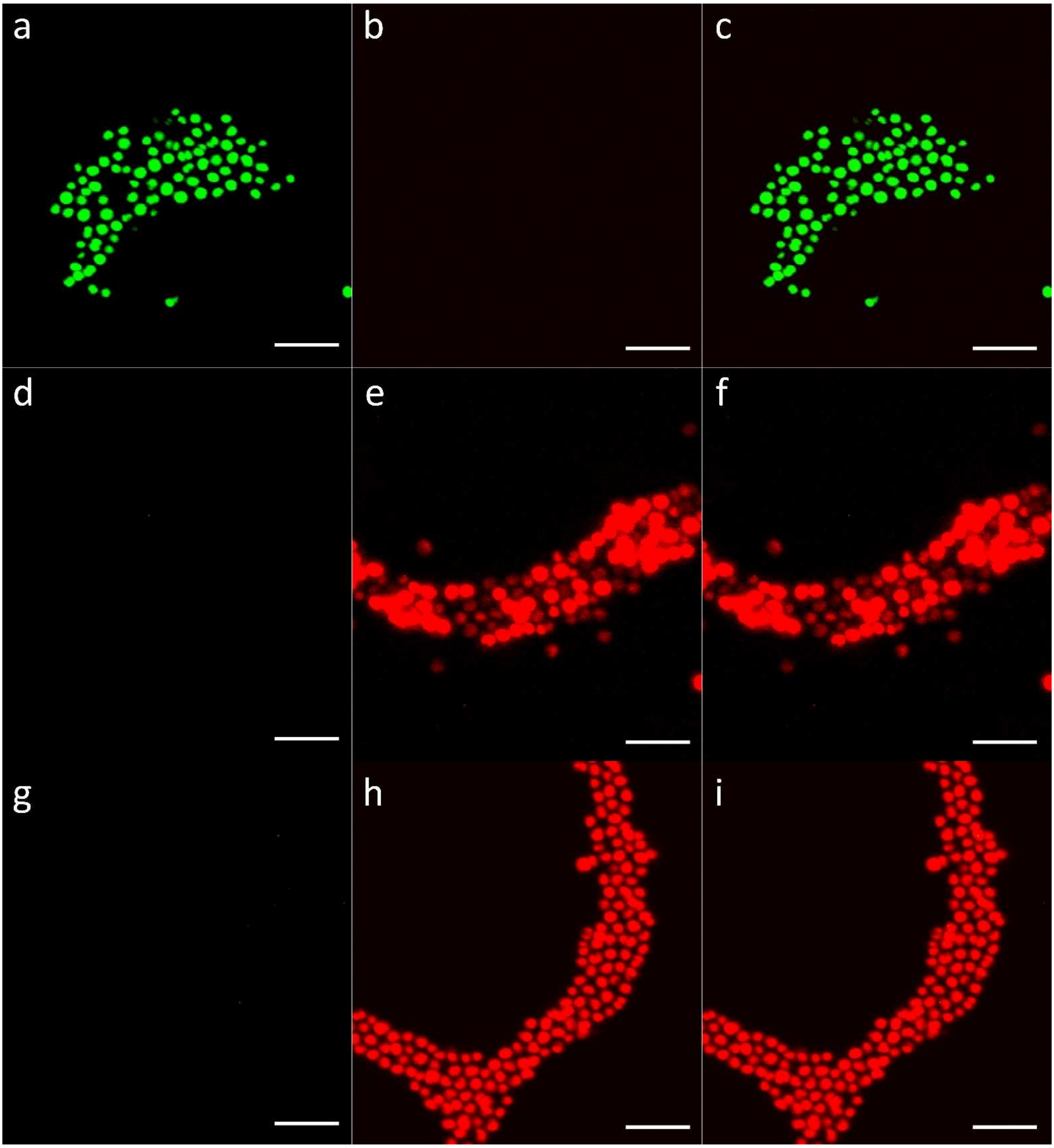

Supplementary Figure 9

Maximum projections of CLSM Z-stacks of viable *S. epidermidis* biofilm stained with SYTO 9 (a-c) or PI (d-f) and **ethanol-fixed biofilm** stained with PI + SYTO 9 (g-i). Green channel (a, d, g), red channel, (b, e, h) and multichannel (c, f, i) projections are presented. The same acquisition and linear post-adjustment settings were applied to all images. Scale bars correspond to 5 µm.

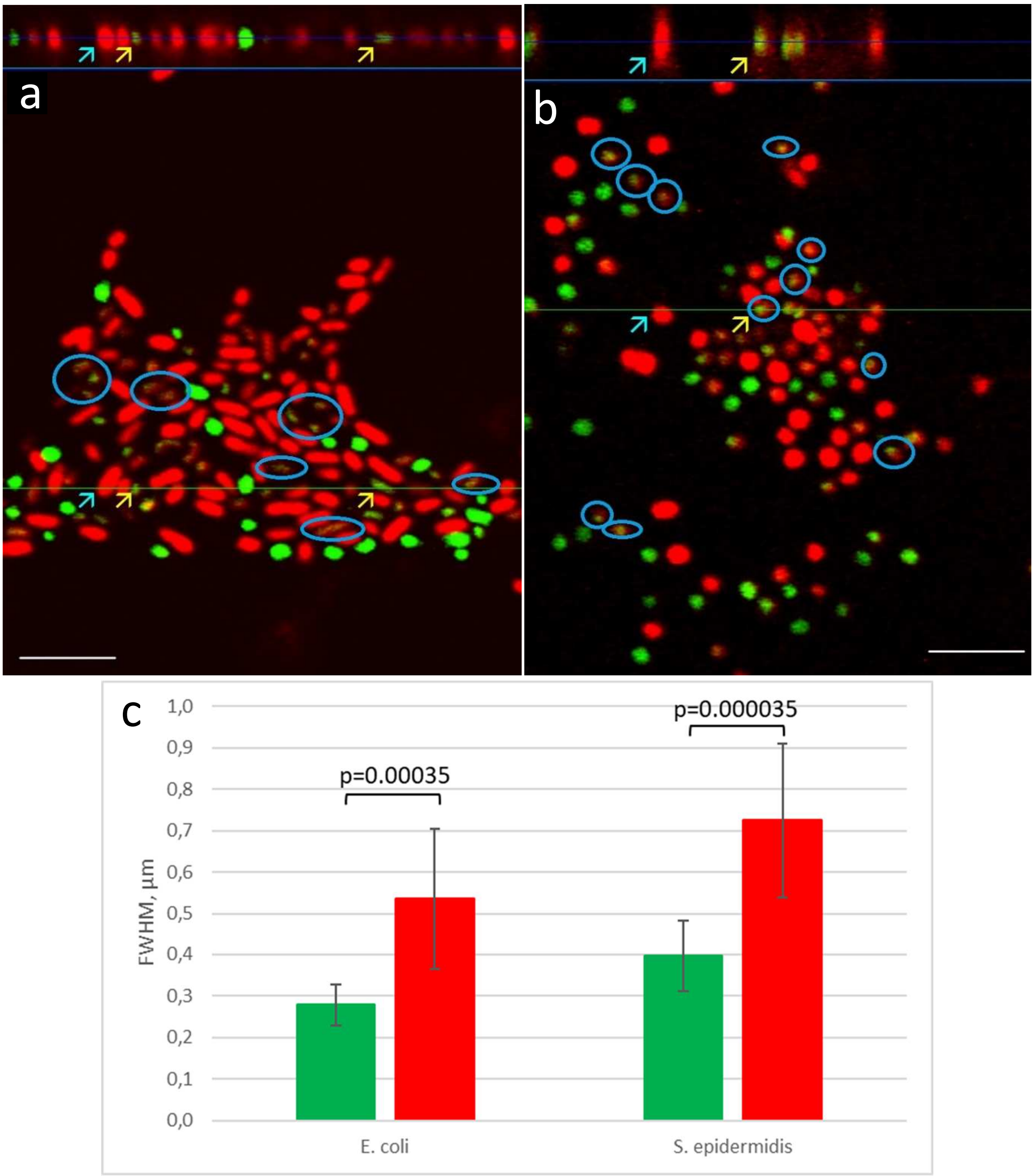

Supplementary Figure 10

Full width at half maximum (FWHM) measurement of non-saturated green SYTO 9 and red PI signals from CLSM cross-sections of *E. coli* (a) and *S. epidermidis* (b) biofilms. Mean and standard deviation of 11 values measured for signals indicated with blue circles on panels a and b are presented (c). Scale bars correspond to 5 µm.

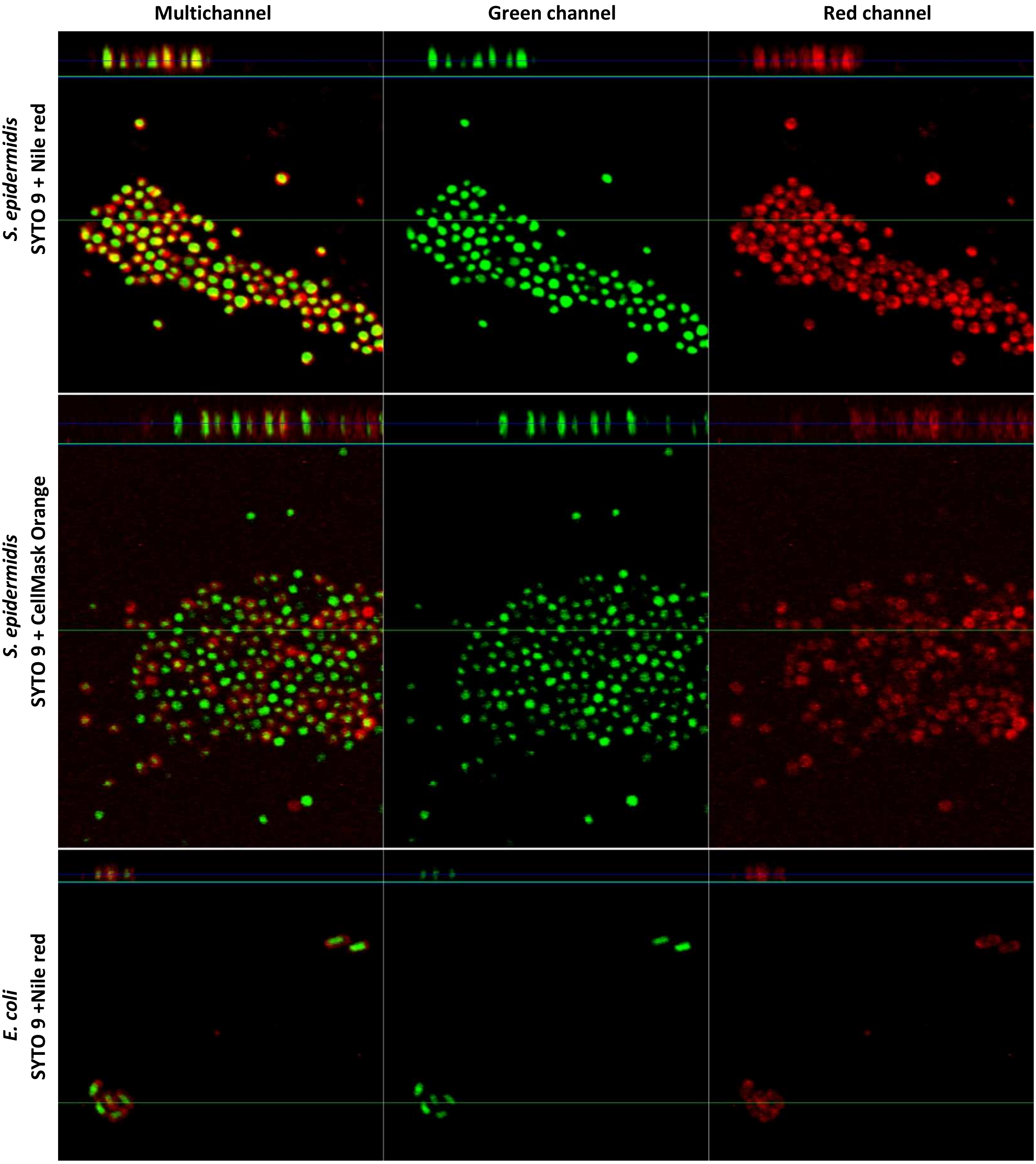

Supplementary Figure 11

Vertical and horizontal CLSM cross-sections of 24 h *S. epidermidis* and *E. coli* biofilms stained with Nile red and CellMask Orange to visualize cell membranes**.** Scale bars correspond to 5 µm.

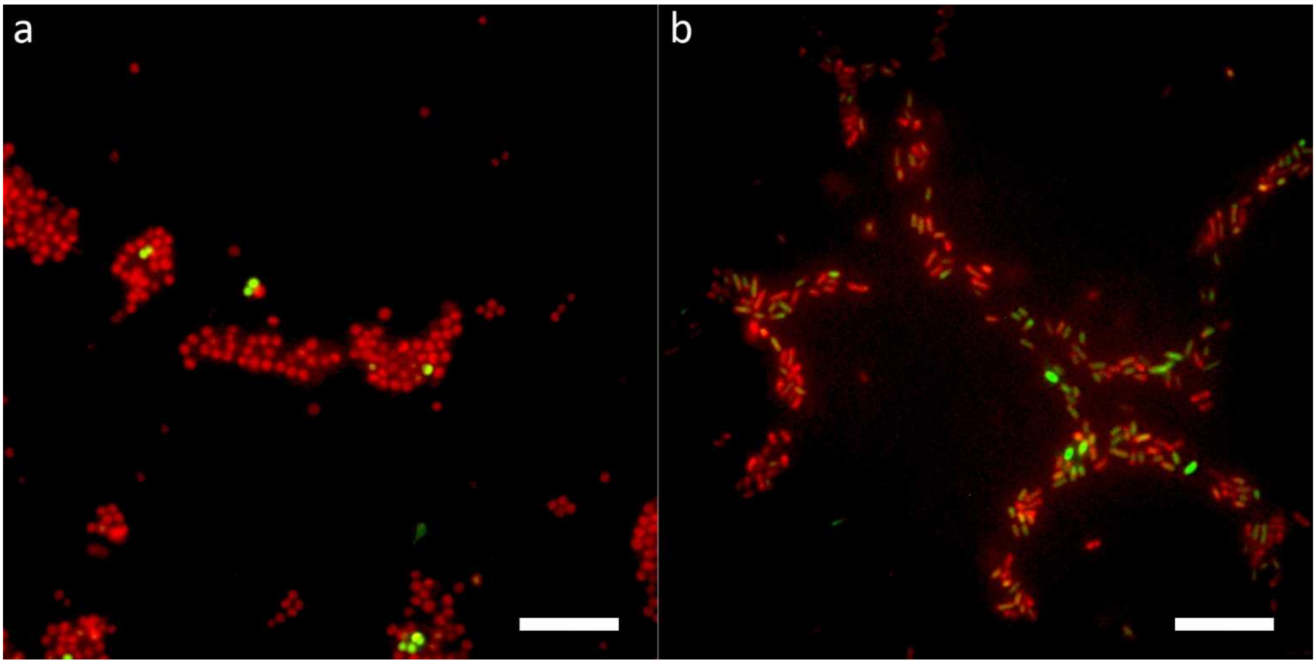

Supplementary Figure 12
Epifluorescence microscopy images 24 h *S. epidermidis* (a) and *E. coli* (b) biofilms co-stained with SYTO 9 and Congo red (CR). Scale bars correspond to 10 µm.

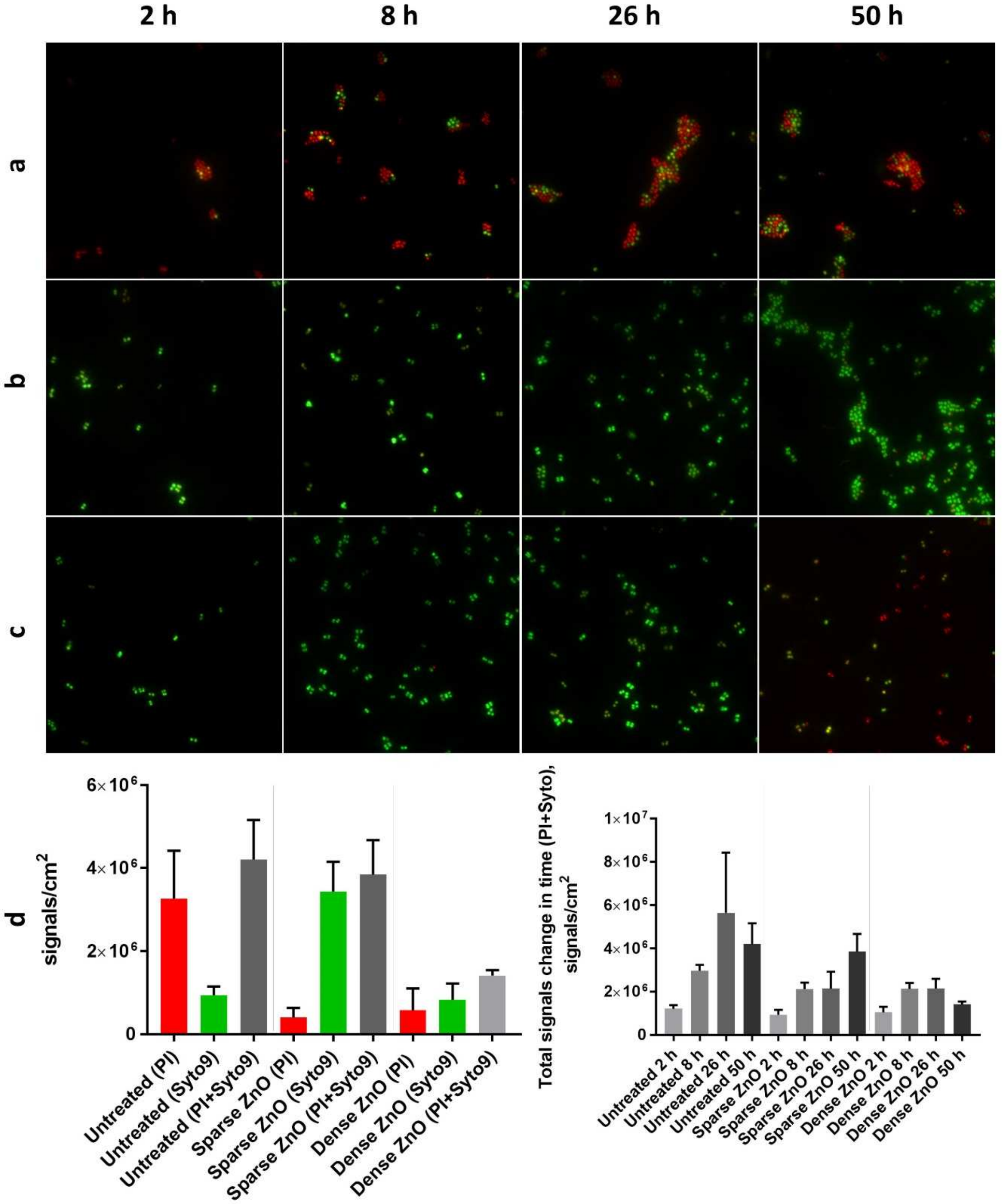

Supplementary Figure 13

Staining of *S. epidermidis* biofilms on glass with propidium iodide (PI) and SYTO 9. *S. epidermidis* DSM20044 biofilm on untreated glass (row a) and on antimicrobial nano-ZnO covered surface described in [51] (row b shows glasses with lower nano-ZnO content and row c shows glasses with higher ZnO content) in PBS stained *in situ* with PI and SYTO 9 and signal counts (row d) of 50 h biofilm (d, left) or total signal count change in time (d, right). Red staining is predominantly observed on non-toxic untreated glass with biofilm specific aggregates formed by tightly bound diplococci while on nano-ZnO antibacterial surfaces biofilm-specific aggregates appear later (b), but stain predominantly green and comprise of loosely bound diplococci and tetrads. Same staining pattern and forming loose diplococci and tetrads is true even in the case on surfaces with even higher nano-ZnO content where biofilm-specific aggregation is not observed during 50 h.
