# Supplementary material for: Propidium iodide staining underestimates viability of adherent bacterial cells
Source: Sci Rep. 2019 Apr 24;9:6483. doi: 10.1038/s41598-019-42906-3 (PMC6482146; doi:10.1038/s41598-019-42906-3)
Supplement: Supplementary file 2 — Supplementary Album 1 [file 41598_2019_42906_MOESM2_ESM.zip › Supplementary Album 1.pdf]

Propidium iodide staining underestimates viability of adherent bacterial cells

Merilin Rosenberg* [1, 2], Nuno F. Azevedo[3], Angela Ivask[1]

[1] Laboratory of Environmental Toxicology; National Institute of Chemical Physics and Biophysics; Akadeemia tee 23, 12618 Tallinn, Estonia
[2] Department of Chemistry and Biotechnology; Tallinn University of Technology; Akadeemia tee 15, 12618 Tallinn, Estonia
[3] LEPABE - Laboratory for Process Engineering, Environment, Biotechnology and Energy; Department of Chemical Engineering; Faculty of Engineering; University of Porto; Rua Dr. Roberto Frias, 4200-465 Porto, Portugal

* rosenbergmerilin@gmail.com; (+372) 5268 384

Supplementary Album 1
Album contains gallery files of single channel and multichannel CLSM Z-stack image series for Figures 3 and 4. Due to oversampling at 0.1 µm interval every 2nd image is presented for *E. coli* and every 3rd image of the stack for *S. epidermidis*.
