# Supplementary figures and images for: Propidium iodide staining underestimates viability of adherent bacterial cells
Source: Sci Rep. 2019 Apr 24;9:6483. doi: 10.1038/s41598-019-42906-3 (PMC6482146; doi:10.1038/s41598-019-42906-3)

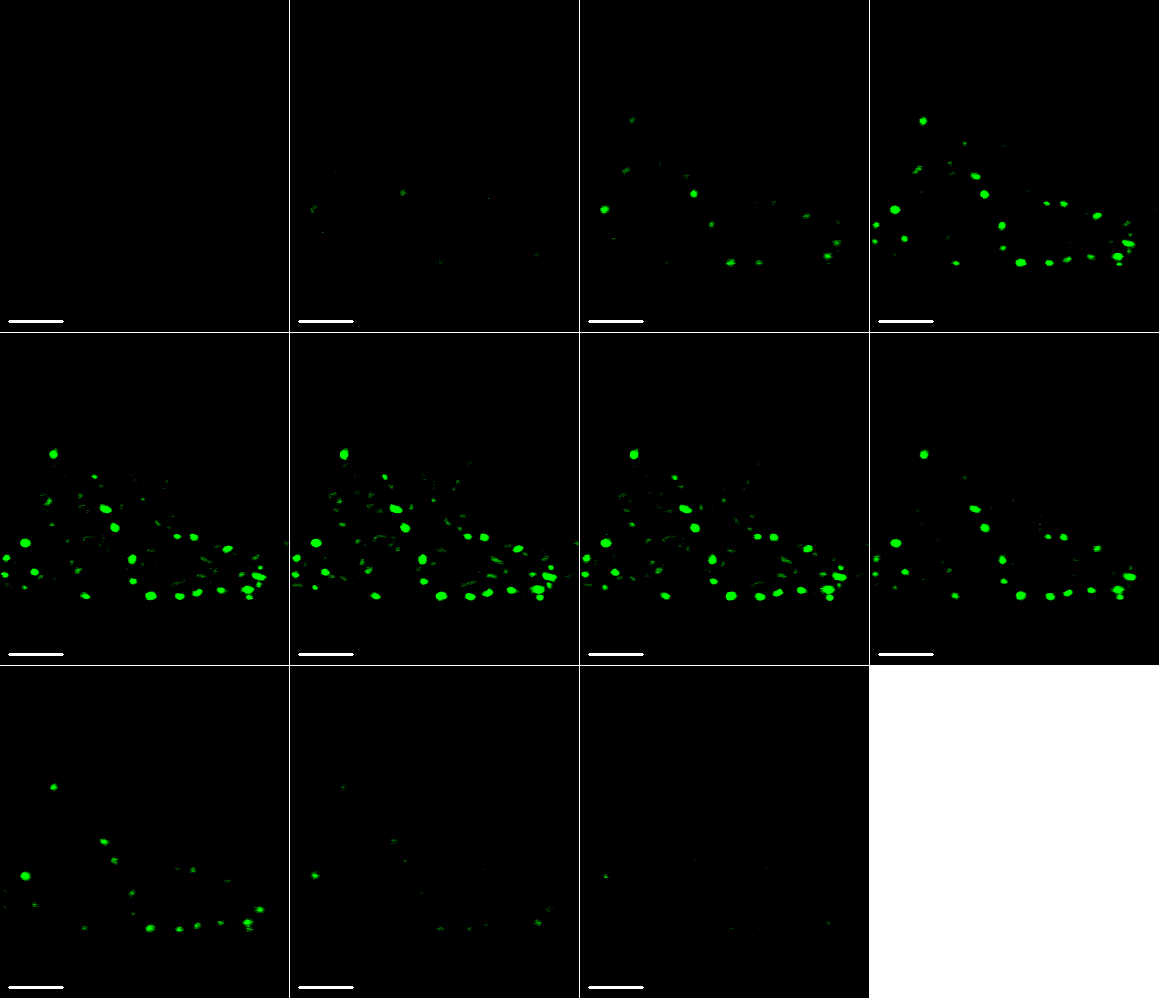

Supplement: Supplementary file 2 — Supplementary Album 1 [file 41598_2019_42906_MOESM2_ESM.zip › E. coli gallery green.tif]

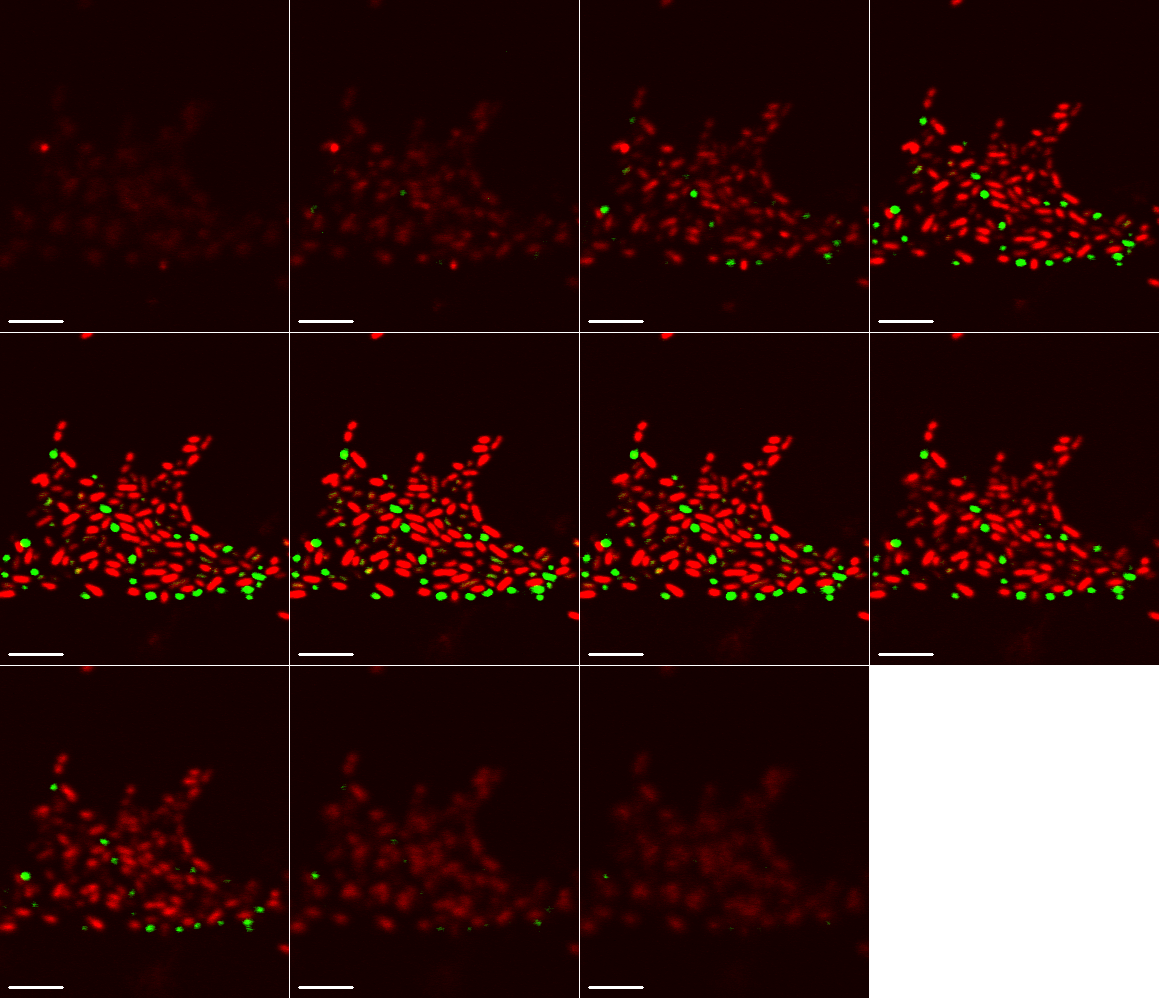

Supplement: Supplementary file 2 — Supplementary Album 1 [file 41598_2019_42906_MOESM2_ESM.zip › E. coli gallery multichannel.tif]

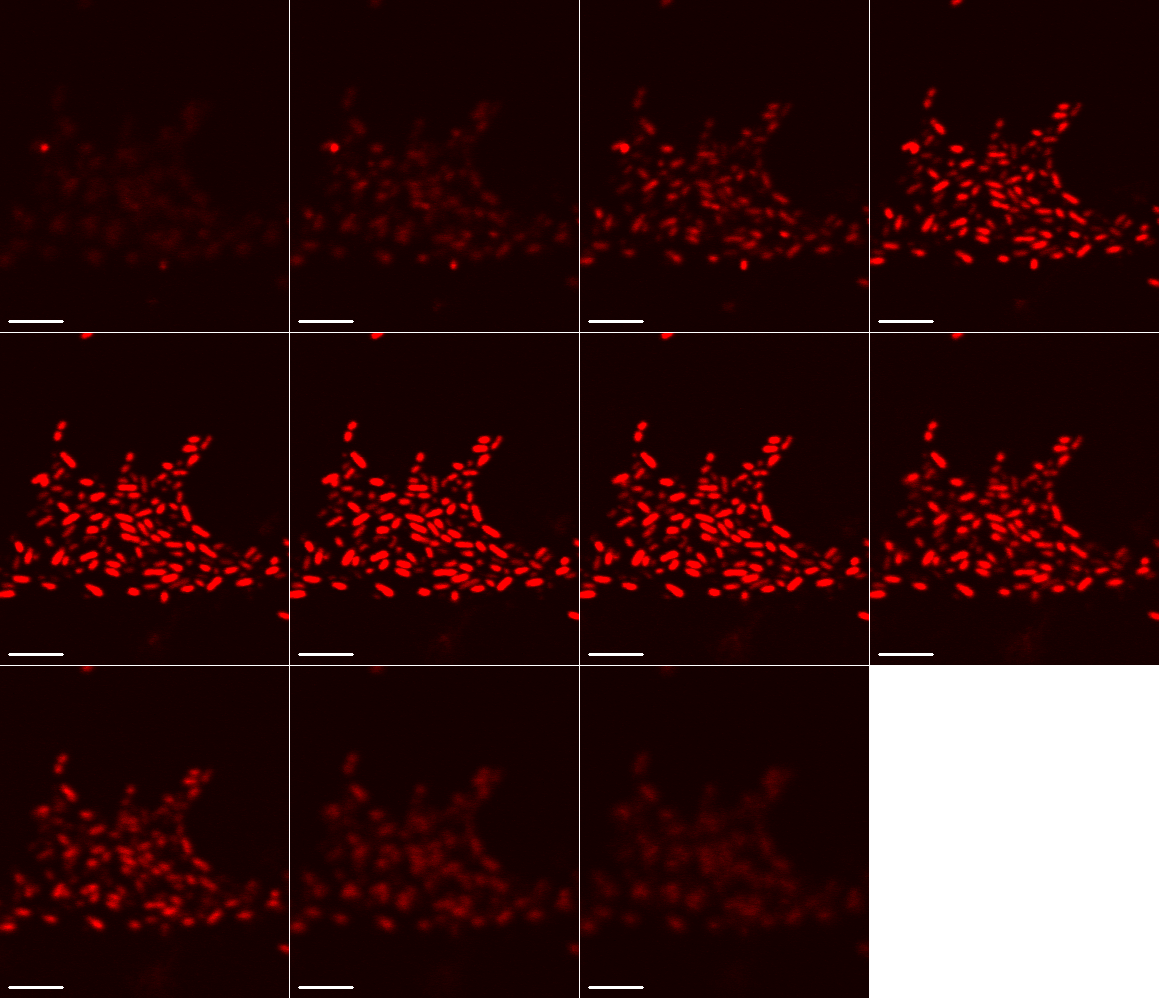

Supplement: Supplementary file 2 — Supplementary Album 1 [file 41598_2019_42906_MOESM2_ESM.zip › E. coli gallery red.tif]

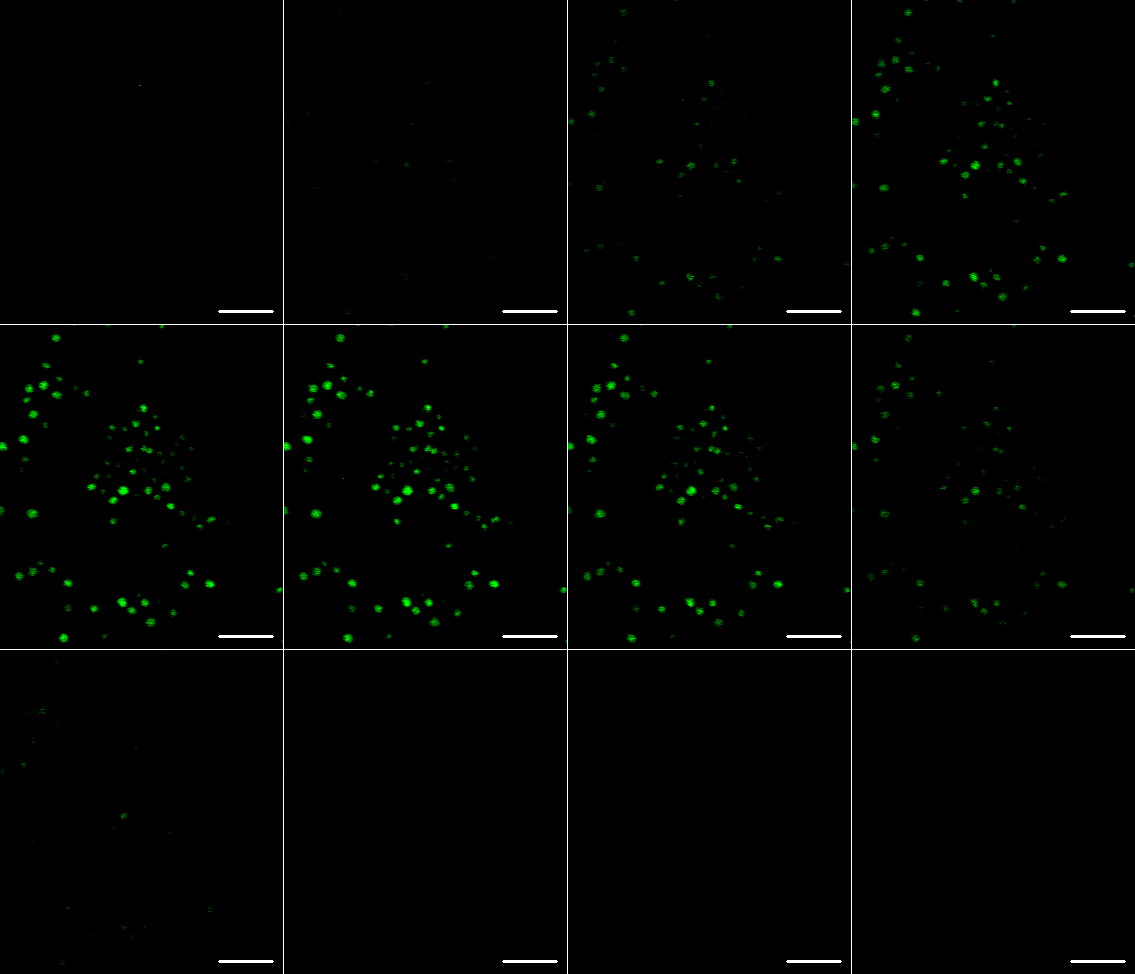

Supplement: Supplementary file 2 — Supplementary Album 1 [file 41598_2019_42906_MOESM2_ESM.zip › S. epidermidis gallery green.tif]

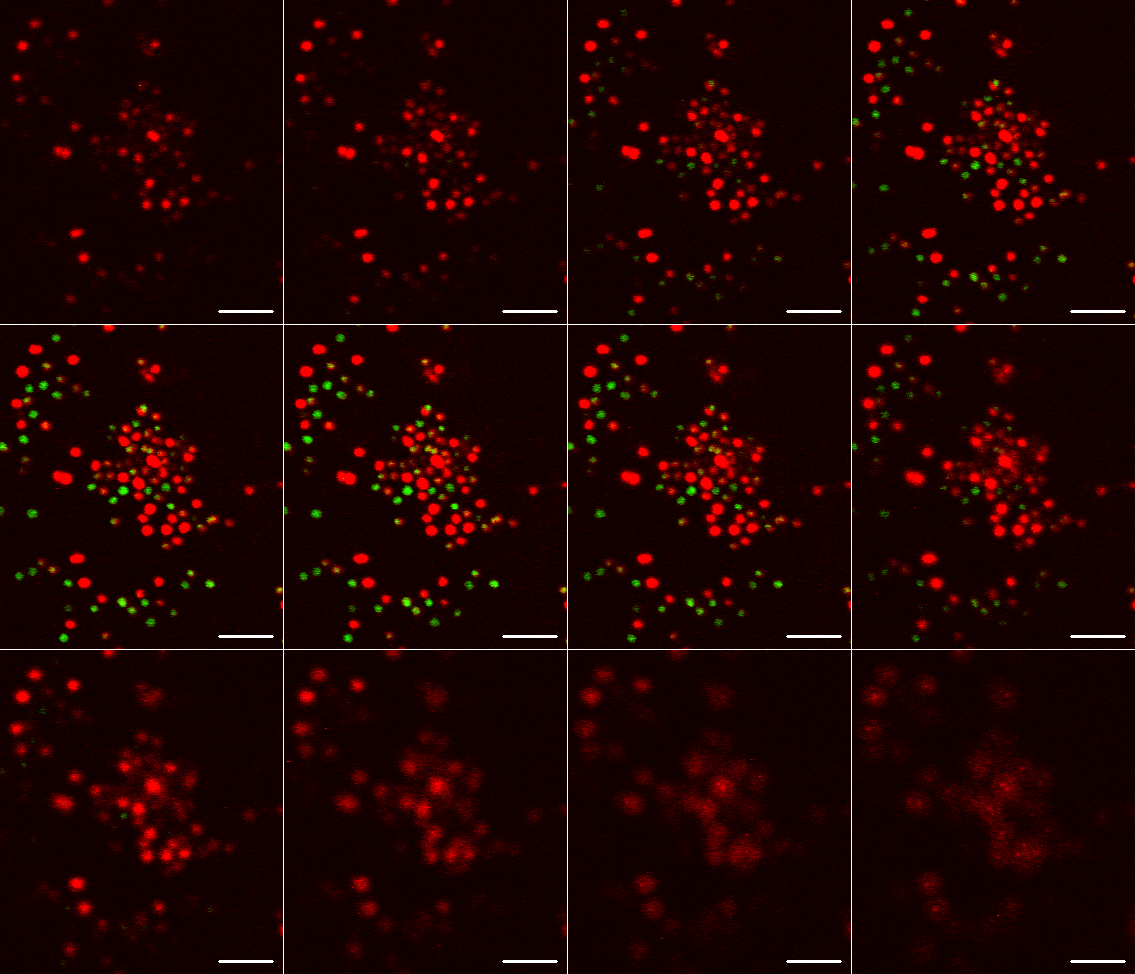

Supplement: Supplementary file 2 — Supplementary Album 1 [file 41598_2019_42906_MOESM2_ESM.zip › S. epidermidis gallery multichannel.tif]

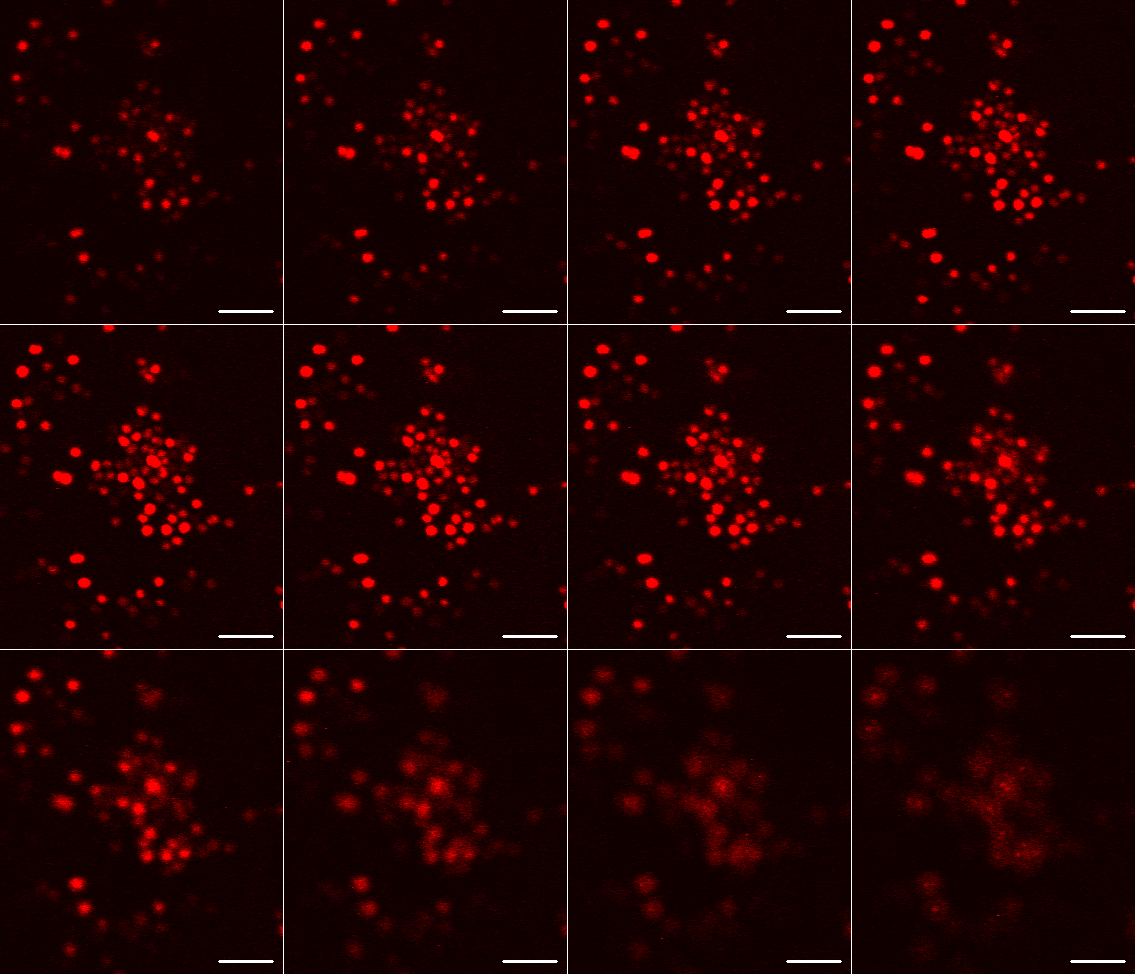

Supplement: Supplementary file 2 — Supplementary Album 1 [file 41598_2019_42906_MOESM2_ESM.zip › S. epidermidis gallery red.tif]
